# System-level consequences of synergies and trade-offs between SDGs: quantitative analysis of interlinkage networks at country level

**DOI:** 10.1007/s11625-022-01109-y

**Published:** 2022-02-27

**Authors:** Jonathan H. P. Dawes, Xin Zhou, Mustafa Moinuddin

**Affiliations:** 1 Centre for Networks and Collective Behaviour, University of Bath, Bath, BA2 7AY UK; 2 Department of Mathematical Sciences, University of Bath, Bath, BA2 7AY UK; 3 Institute for Global Environmental Strategies (IGES), 2108-11 Kamiyamaguchi, Hayama, Kanagawa 240-0115 Japan

**Keywords:** Sustainable Development Goals, Network science, Mathematical modelling, Hierarchy, Trade-offs

## Abstract

The Sustainable Development Goals (SDGs) present a complex system of 17 goals and 169 individual targets whose interactions can be described in terms of co-benefits and trade-offs between policy actions. We analyse in detail target-by-target interlinkage networks established by the Institute for Global Environmental Strategies (IGES) SDG Interlinkages Tool. We discuss two quantitative measures of network structure; the leading eigenvector of the interlinkage networks (‘eigencentrality’) and a notion of hierarchy within the network motivated by the concept of trophic levels for species in food webs. We use three interlinkage matrices generated by IGES: the framework matrix which provides a generic network model of the interlinkages at the target level, and two country-specific matrices for Bangladesh and Indonesia that combine SDG indicator data with the generic framework matrix. Our results echo, and are confirmed by, similar work at the level of whole SDGs that has shown that SDGs 1–3 (ending poverty, and providing food security and healthcare) are much more likely to be achieved than the environmentally- related SDGs 13–15 concerned with climate action, life on land and life below water. Our results here provide a refinement in terms of specific targets within each of these SDGs. We find that not all targets within SDGs 1–3 are equally well-supported, and not all targets within SDGs 13–15 are equally at risk of not being achieved. Finally, we point to the recurring issue of data gaps that hinders our quantitative analysis, in particular for SDGs 5 (gender equality) and 13 (climate action) where the huge gaps in indicator data that mean the true nature of the interlinkages and importance of these two SDGs are not fully recognised.

**Supplementary Information:**

The online version contains supplementary material available at 10.1007/s11625-022-01109-y.

## Introduction

The 2030 Agenda for Sustainable Development is ‘a plan of action for people, planet and prosperity’ UN General Assembly (2015) that sets out ambitions in 17 Sustainable Development Goals (SDGs) covering the broad areas of humanity’s activity, relationships, and the global environment. As the Resolution adopted by the General Assembly in 2015 goes on to set out, at the coarsest-grained level the SDGs can be summarised as encompassing people, planet, prosperity, peace, and partnerships. At the finest-grained level the SDGs consist of 169 targets distributed over the 17 Goals and monitored by 231 unique statistical indicators (SDG Indicators 2021).

The existence of trade-offs, synergies and co-benefits between the 17 Goals and 169 Targets that comprise the 2030 Agenda for Sustainable Development have been highlighted by many authors not least the commentary by ICSU (2015) and subsequent analysis by Le Blanc (2015). Since then, broadly speaking, detailed work has focussed either on data-driven analyses of correlations between indicators related to SDGs and individual targets (Sachs et al. 2019, 2020), or on expert opinion and literature reviews surveying the likely extent and direction of interlinkages between SDGs and their separate targets (van Soest et al. 2019; Pham Truffert et al. 2020a, b; Dawes 2020).

However, there is a need to go further than simply the construction of interlinkage networks, and to develop methodologies that enable policy implications to be drawn in straightforward and robust ways from the structure of the interlinkages. Most obviously this concerns prioritisation within the SDG network: for a particular region or country, within its limits of resources and capacities, which areas of the 2030 Agenda should be given a particular emphasis? The Asia-Pacific region provides a very good example of the need for prioritisation. Even though the region has made remarkable progress in many SDGs, particularly Goal 4 (Education) and Goal 7 (Energy), the region will not achieve any of the 17 Goals with business-as-usual policies (ESCAP 2020). Even before the COVID-19 pandemic unfolded, the decade of action for achieving the SDGs in Asia-Pacific was already in crisis. Policy makers in the region therefore have a strong current practical need to accelerate progress on the SDGs through effective prioritisation.

In this paper we analyse data and interlinkages for two specific countries in the Asia-Pacific region: Bangladesh and Indonesia. Our selection of Bangladesh and Indonesia for the empirical analysis is based on the experience and on-going activities of the Institute for Global Environmental Strategies (IGES) in these two countries which we briefly summarise here; additional background is provided in second section. Bangladesh and Indonesia are developing countries in Asia that share a high vulnerability to climate change while being in other respects very different. For example Indonesia’s economy is nearly six times the size of Bangladesh’s, and the level of GDP per capita in Indonesia was, in 2018, more than three times that of Bangladesh. Both countries have responded actively to the 2030 Agenda; they have made great efforts to integrate the SDGs into national development planning, have established inter-ministerial coordination mechanisms, and have developed national systems to monitor SDG progress.

In this paper we apply recently-developed quantitative methods from network science to examples of interlinkage networks to draw system-level conclusions. We describe interlinkages by an adjacency matrix *A* that describes the set of directed influences between individual targets. We define the matrix *A* using the convention that the element $A_{ij}$ describes the influence of target *j* on target *i*, i.e. $j \rightarrow i$.

The mathematical techniques that we describe below can be applied to any interlinkage matrix. In this paper we illustrate the analyses using data from the SDG Interlinkages Tool developed by the IGES (Zhou et al. 2021). The IGES methodology (Zhou and Moinuddin 2017), described in more detail in “Data” section below, proceeds in two distinct stages. First, a generic *framework matrix* is constructed. The framework matrix describes which interactions between targets are plausible and could arise from natural, or typical, choices in public policy. The entries in the framework matrix are therefore either zero or one, indicating the absence or presence of a potential interlinkage. The framework matrix is also referred to as the ‘IGES generic model’ as it is an unweighted adjacency matrix (i.e. it has entries that are either zero or one but no intermediate values), built based on causalities deduced through a combination of a comprehensive literature review, expert judgement and stakeholder consultations.

In the second stage of the IGES methodology, the framework matrix is refined into a country-specific model through quantitative estimation of the relative strengths of the interlinkages for that country. In other words, the zeros in the framework matrix are kept as zeros but the ones are replaced by correlation coefficients (in the range $-1$ to 1) that provide a quantitative estimation of the strength (weak vs. strong) and nature (positive or negative) of each interlinkage in a way that is directly relevant to that specific country. The most natural source for this estimation is a correlation analysis of the country-level time-series data for indicators for each pair of targets. In many cases such historic data is available over the full two decades of the period 1990–2019 which gives us some confidence in the historic alignment, as a proxy for policy interaction, between each pair of targets.

Since the interlinkage matrices share a common underlying structure, it is of interest to understand the underlying implications of these two separate parts to the IGES methodology. That is, the structure imposed by the framework matrix itself, and then subsequently the results for different countries, where differences must be due to the historic correlations, indicating differences in policy coherence or the policy instruments that are available. Our preferred approach to these structural questions is to compute the eigencentrality of the individual targets which we can interpret as a measure of the support that each target receives from the remainder of the network, and therefore, as we explain later, how rapidly progress on that target is likely to be made. We illustrate this approach through two country-specific cases.

Our second quantitative methodology is to develop further the notion of hierarchy in directed networks proposed by MacKay et al. (2020). This methodology complements the analysis developed in the context of the Global Sustainable Development Report (Pham Truffert et al. 2020a, b), abbreviated to GSDR, which focussed on comparisons of in-degree and out-degree between network nodes (i.e. the number of incoming and outgoing directed edges for each node). In the GSDR, nodes with high in-degree are referred to as ‘buffers’, while those with high out-degree are called ‘multipliers’ since progress on a target with a high out-degree is likely to generate wider effects on other targets that are ‘downstream’ of it. As we discuss further below, our analyses extend this idea by considering concepts that take a holistic view of the network rather than considering the network node by node.

In summary, the paper makes new contributions both by developing appropriate network science tools, and also by applying them to specific interlinkage networks at country-level, allowing detailed implications for policymaking to be drawn out.

The structure of the remainder of the paper is as follows. In second section we discuss the data sources and methods that our analysis is based on. Third section contains the results of those analyses and initial comments. Fourth section contains a discussion of the analyses together and presents our conclusions.

## Materials and methods

### Development context

To introduce the context for our subsequent analysis we briefly summarise the background to the SDGs within Bangladesh and Indonesia and the level of recent engagement with 2030 Agenda.

#### Bangladesh

The Government of Bangladesh embraced the 2030 Agenda and the SDGs with great enthusiasm. Bangladesh’s performance in implementing the Millennium Development Goals (MDGs) shows that impressive progress was achieved in areas such as poverty reduction, gender equality and universal primary education, but there are still unfinished tasks and remaining challenges in employment generation, primary school completion and adult literacy rate, decent wage employment for women, skilled health professionals, and forest area coverage (GED 2015b, 2016b). Building on the progress inspired by the MDGs, policymakers in Bangladesh started to plan the country’s implementation of the SDGs from a very early stage. The 2030 Agenda receives the highest political support from the Prime Minister’s Office in Bangladesh, and an inter-ministerial committee coordinates the implementation and review of the SDG implementation processes. At the time the 2030 Agenda was being developed, Bangladesh was working on its 7th Five Year Plan (FYP) for 2016–2020, allowing the 7th FYP to be aligned with the SDGs (GED 2015a, 2016a; GPRB 2017a)

The government also developed a detailed SDG financing strategy (GED 2017c) and produced consultation documents on how to make the country’s SDG approach more integrated by taking account of the linkages among the SDG goals and targets and by identifying the lead and co-lead agencies for each of the SDG targets GED (2016a). Bangladesh submitted its first Voluntary National Review (VNR) in 2017, and the second one in 2020 (GPRB 2017a, 2020). The country has identified a list of 40 priority indicators, adopted its SDG Action Plan, and developed a tracker to monitor the progress of SDG implementation (GED 2017b; GPRB 2020). Among the priority indicators, 39 are considered to be important for localising the SDGs and for creating synergistic effects on other SDG targets and indicators, while the last one reflects the principle of leaving no one behind, taking into consideration the specific context of each of the 64 districts of the country. While the COVID-19 pandemic has affected the overall implementation of the SDGs, Bangladesh also intends to address existing inequities while addressing the pandemic (GPRB 2020).

#### Indonesia

Indonesia considers the 2030 Agenda as an opportunity for the country’s sustainable, inclusive, prosperous and resilient future. Mandated by a Presidential Regulation of 2017, Indonesia has aligned its Long Term and Medium Term National Development Plans 2020–2034 (BAPPENAS 2007; BAPPENAS 2018) with the SDGs. Its planning and implementation of the SDGs are coordinated by the Indonesian Ministry of National Development Planning (BAPPENAS), where the National SDGs Secretariat has been established. The country’s SDG implementation is guided by the National SDGs Road Map 2017–2030, National Action Plan for the SDGs, and the Regional Action Plans for the provinces (BAPPENAS 2019a; Reagan 2019). Indonesia has submitted three VNRs so far, in 2017, 2019 and 2021, respectively BAPPENAS (2017, 2019a, 2021).

The 2021 VNR discusses the national SDGs planning and policy processes, progress made so far, challenges ahead especially in the context of the COVID-19 pandemic, and the future of progress on the SDGs. The 2021 VNR highlights several areas, including social protection, healthcare, disaster resilience, and economic recovery where continued efforts are needed. Referencing the SDG interlinkage analysis from the National SDGs Road Map based on the IGES SDG Interlinkages methodology (Zhou and Moinuddin 2017; Zhou et al. 2021), the 2021 VNR stresses how the COVID-19 recovery policy priorities in the above-mentioned areas are relevant for the achievement of the SDGs in Indonesia. Governance, stakeholders engagement and financing for SDGs are still some of the major challenges facing SDG implementation in Indonesia. The 2021 VNR also highlights the principle of leaving no one behind as a cornerstone of Indonesia’s SDG implementation strategy (BAPPENAS 2021).

### Data

In this paper we focus on three specific interlinkage networks produced by IGES. Detailed description of the IGES methodology is presented elsewhere (Zhou and Moinuddin 2017; Zhou et al. 2021), but a brief summary is appropriate here to draw out the notable features that ensure it is both robust and grounded in data. The IGES methodology considers individually all 169 targets that comprise 2030 Agenda. For each Goal there are targets of two distinct kinds articulated in the 2030 Agenda: the first kind are outcome-related targets, labelled numerically, while the second are the ‘means of implementation’ (MoI) targets that describe the necessary steps that must be taken to achieve the desired outcomes. The MoI targets for each SDG are labelled alphabetically. To illustrate the contrast between the two kinds of target, compare target 5.1 *End all forms of discrimination against women and girls everywhere* with the MoI target 5.b *Enhance the use of enabling technology, in particular information and communications technology, to promote the empowerment of women*. Target 5.b describes how progress towards target 5.1 can be achieved. The IGES analysis considers both kinds of targets. There are generally more outcome-related targets than there are MoI targets for each Goal, as summarised in Table 1. In addition the targets within SDG 17 are in a sense all concerned with the means of implementation of 2030 Agenda. In accordance with (UN General Assembly 2015, paragraph 40) we treat all targets equally in our analysis.

**Table 1:** The 17 Sustainable Development Goals and the number of individual targets associated with each Goal. Targets are separated into those that are outcome-related and those designated as the ‘means of implementation’ (MoI). These numbers are shown separately for each SDG. For example SDG 3 has nine outcome-related targets plus four MoI targets, making 13 targets in total for SDG 3. Overall there are 126 outcome-related targets and 43 MoI targets, making 169 in total

	Number of targets	
SDG number and short title	Outcome-related	Means of implementation
1. No poverty	5	2
2. Zero hunger	5	3
3. Good health and well-being	9	4
4. Quality education	7	3
5. Gender equality	6	3
6. Clean water and sanitation	6	2
7. Affordable and clean energy	3	2
8. Decent work and economic growth	10	2
9. Industry, innovation and infrastructure	5	3
10. Reduced inequalities	7	3
11. Sustainable cities and communities	7	3
12. Responsible consumption and production	8	3
13. Climate action	3	2
14. Life below water	7	3
15. Life on land	9	3
16. Peace, justice and strong institutions	10	2
17. Partnerships for the goals	19	0

**Fig. 1:**
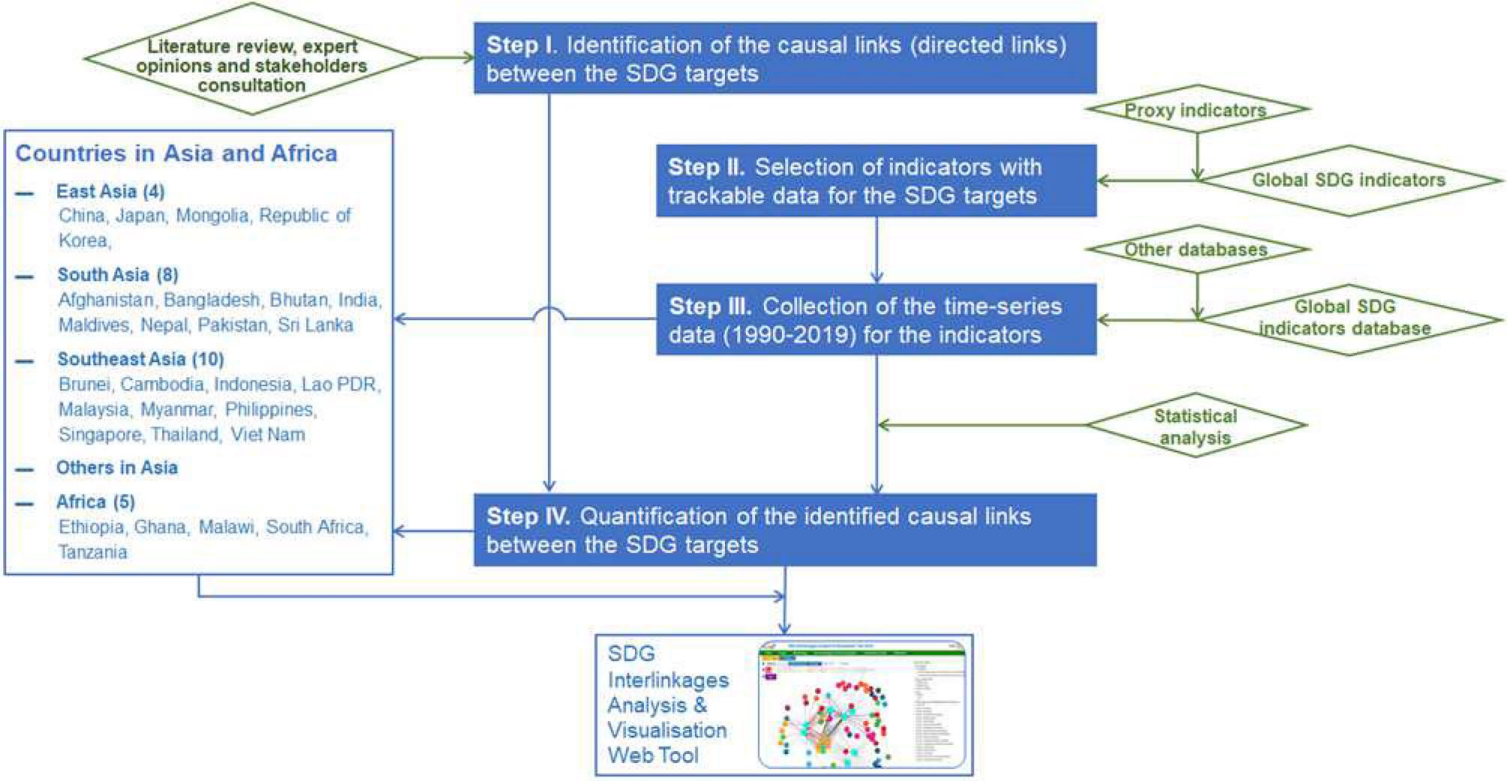
Flowchart of the IGES methodology for SDG interlinkage analysis. Source: Zhou et al. (2021)

The IGES methodology is a four-step process (see Fig. 1) that was first developed in 2017 and later updated to reflect new developments and tailored to specific case studies. The four steps are as follows. Step I is an identification of the causal links (directed network edges) between the SDG targets. This was conducted based on a comprehensive review of relevant references on SDG interlinkages, including the working documents provided by relevant international consultation processes on SDG indicators (International Council for Science 2015, IAEG-SDG 2015; SDSN 2015), and other publications on specific goals and their interactions with other SDGs, for example Goal 6 (ESCAP 2017), Goals 2, 3, 7 and 14 (ICSU 2017), and Goal 12 (Coopman et al. 2016).

The literature review has been gradually advanced to include recent developments, e.g. the report by the Interlinkages Working Group of the IAEG-SDGs (IAEG-SDG 2019), and academic work (through a systematic review of literature from the ScienceDirect database). For a couple of case studies on specific topics (e.g. SDGs at the river basin scale, national strategy on sustainable consumption and production, SDGs localisation, and urban–rural linkages), expert opinions and stakeholder consultation were conducted to complement the literature review and reflect the context of the topic (Baffoe et al. 2021; King et al. 2020; ‘Luanhe Living Lab’ project team 2020). The output of Step 1 is a non-symmetric binary (0 or 1) $169 \times 169$ matrix where ‘1’s indicate the presence of causal links between the pair of targets and ‘0’s indicate that there is no casual link. The binary matrix defines the structure of the SDG interlinkage network but is not specific to any one country. We refer to the binary matrix *A* as the ‘generic model’ or ‘framework matrix’, and the entry $A_{ij}$ describes the possibility, or absence, of an influence from target *j* to target *i*.

In Step II sets of indicators are selected for which there are sufficient trackable data for the SDG targets based on the Global SDG Indicators. When indicators or relevant data are not available, other proxy indicators (e.g. the World Bank’s World Development Indicators) were used (see Zhou et al. 2021, for details). Step III is the collection of the time-series data (for the period 1990–2019) of the indicators for 27 countries in Asia and Africa. In Step IV the strength of the causal links are computed based on the Pearson correlation coefficients of the indicator-level time-series data, for each of the 27 countries. These correlation coefficients then become the elements of the country-specific adjacency matrix *A* but are computed only where a potential causal link is considered to exist, i.e. where the framework matrix has a ‘1’. As a result, the generic model and the quantitative models for each of the 27 countries share the same underlying network structure but their non-zero entries differ.

If the framework matrix has causal links in both directions between two targets *i* and *j*, i.e. $A_{ij}=1$ and $A_{ji}=1$, then the quantitative model for the country has the same value for the elements $A_{ij}$ and for $A_{ji}$, i.e. the link is considered to be bi-directional. If the causal link in the framework matrix is only in one direction, say from target *j* to target *i*, but not from *i* to *j*, then $A_{ij}$ in the country matrix will be set to the (nonzero) value of the correlation coefficient but the element $A_{ji}$ will be set to zero as the causal link has been determined to be only in one direction.

**Fig. 2:**
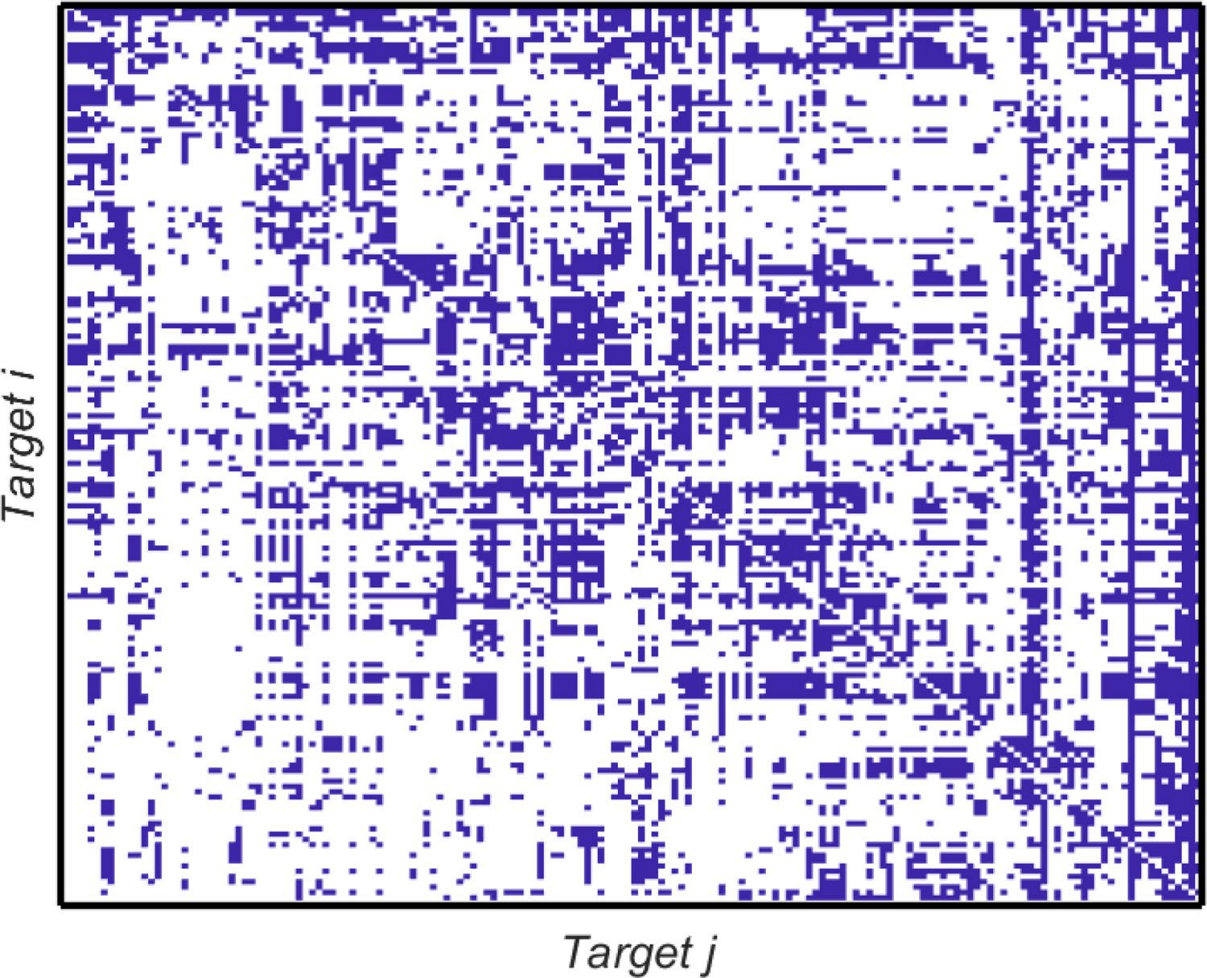
The framework matrix *A* developed in the IGES Interlinkages Tool. Entry $A_{ij}$ indicates the effect of target *j* (labelling the columns of the matrix) on progress on target *i* (labelling the rows of the matrix). Entries in the matrix are either one (dark blue) or zero (white), indicating the potential presence or absence of that interlinkage between the relevant pair of targets. Note that the matrix is presented transposed compared to those in the IGES reports (Zhou and Moinuddin 2017; Zhou et al. 2021)

The framework matrix developed within the IGES Interlinkages Tool shares similar overall structural features with the Goal-level interaction matrices analysed in previous work (Dawes 2020, 2021). In particular the framework matrix is sparser in the lower left and along the bottom than it is across the top of the matrix and on the right hand side. This indicates, firstly, that the influence of targets from higher-numbered Goals on targets in lower-numbered Goals is greater than the other way around. Secondly, it shows that some targets in high-numbered Goals are considered potentially to impact almost every other target.

The targets with a particularly high out-degree (number of other targets that they directly influence) are targets 17.9 (‘International support for sustainable development capacity building in developing countries’) and 17.18 (‘Capacity building for developing countries in data availability’) which have an out-degree of 168, i.e. they are considered potentially to influence every other target, and target 16.6 (‘Develop accountable institutions’) which has an out-degree of 145. These three targets show up as vertical lines on the right of Fig. 2. The denser nature of the matrix on the right-hand side illustrates the general observation that SDG 17 should be a key influence driving progress across the whole of the 2030 Agenda. Finally we note also that the framework matrix does not consider a target to be linked to itself: all the diagonal entries of the framework matrix are zero by construction.

**Fig. 3:**
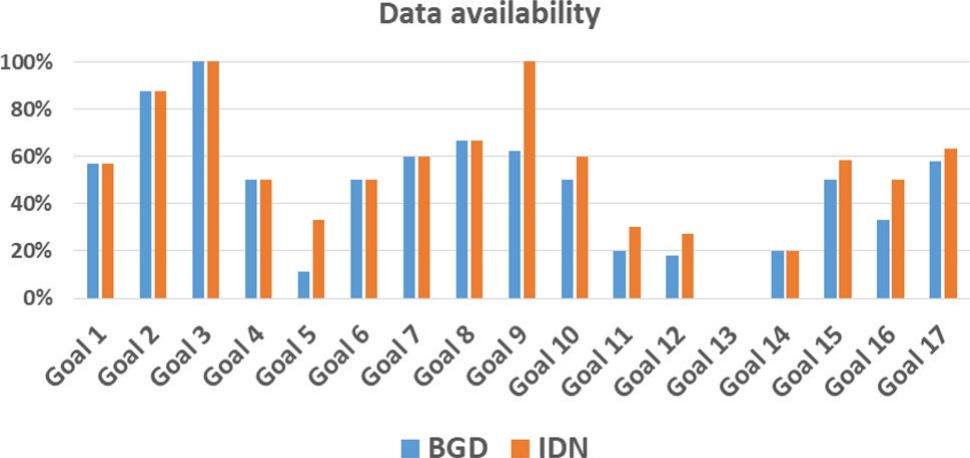
Data availability for Indonesia and Bangladesh, expressed as a percentage of the available indicator time series for the period 1990–2019, for each SDG

**Fig. 4:**
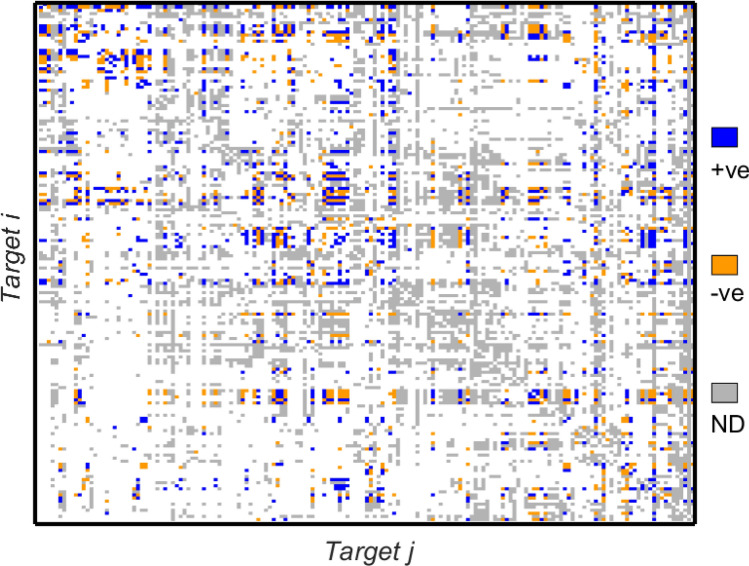
The target-level interaction matrix derived for Indonesia (IDN). Entries are colour-coded as blue (‘+ve’—positive), orange (‘-ve’—negative) or grey (‘ND’—No Data). Grey entries indicate where the generic framework matrix has a 1 entry but missing data prevents calculation of the correlation coefficient for the pair of targets

**Fig. 5:**
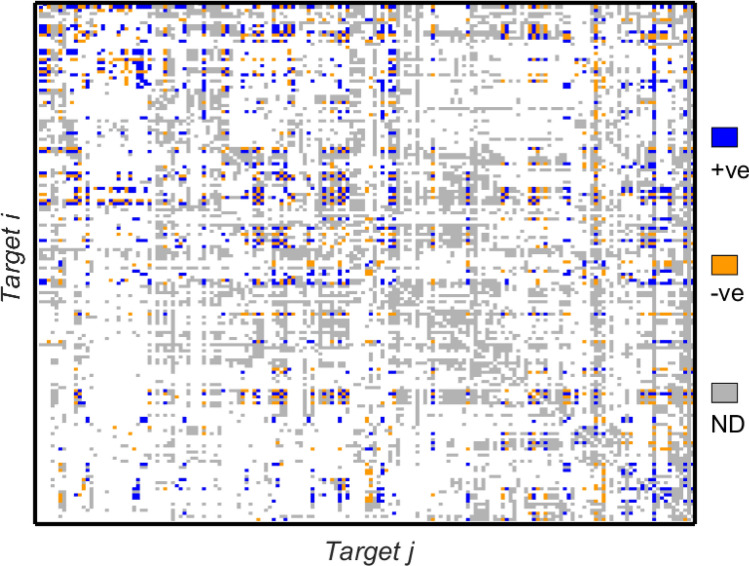
The target-level interaction matrix derived for Bangladesh (BGD). As in Fig. 4, entries are colour-coded as blue (‘+ve’—positive), orange (‘-ve’—negative) or grey (‘ND’—No Data). Grey entries indicate where the generic framework matrix has a 1 entry but missing data prevents calculation of the correlation coefficient for the pair of targets

We turn now to the country-specific data for Indonesia (IDN) and Bangladesh (BGD). A key challenge for both countries is the availability of indicator time series for indicators related to each target. Figure 3 summarises the indicator data that is available for Indonesia and Bangladesh for each SDG. While the general pattern across SDGs is extremely similar for the two countries, the situation for Indonesia is slightly better overall. Indicator data relating to indicators for SDG 13 is completely missing, while data for SDG 5 and SDGs 11–14 are significantly lacking which implies that our conclusions in relation to these SDGs may be subject to greater uncertainty than for others.

For both countries data gaps mean that a significant number of entries in the network matrix are not available. It is of interest to note that where data is available there are significant numbers of negative interactions (shown in orange in the figure). The sign and strength of entries are determined from correlations between time series for indicators corresponding to the pair of targets in question. Since data are not available for every target, a substantial fraction of the possible entries identified in Fig. 2 are not able to be computed. The matrices $A_\mathrm {BGD}$ and $A_\mathrm {IDN}$ for which the interaction matrices are summarised in Figs. 4 and 5. Where data is available, the correlation analyses yield values in the range $-1,\ldots , +1$. Since many of the computed values are close to the extreme values of $\pm 1$ for ease of illustration in Figs. 4 and 5 to bring out the contrast with the missing data we do not show a continuous colour scale but instead colour all the positive entries blue and all the negative entries orange in order to make the plot as clear as possible.

**Fig. 6:**
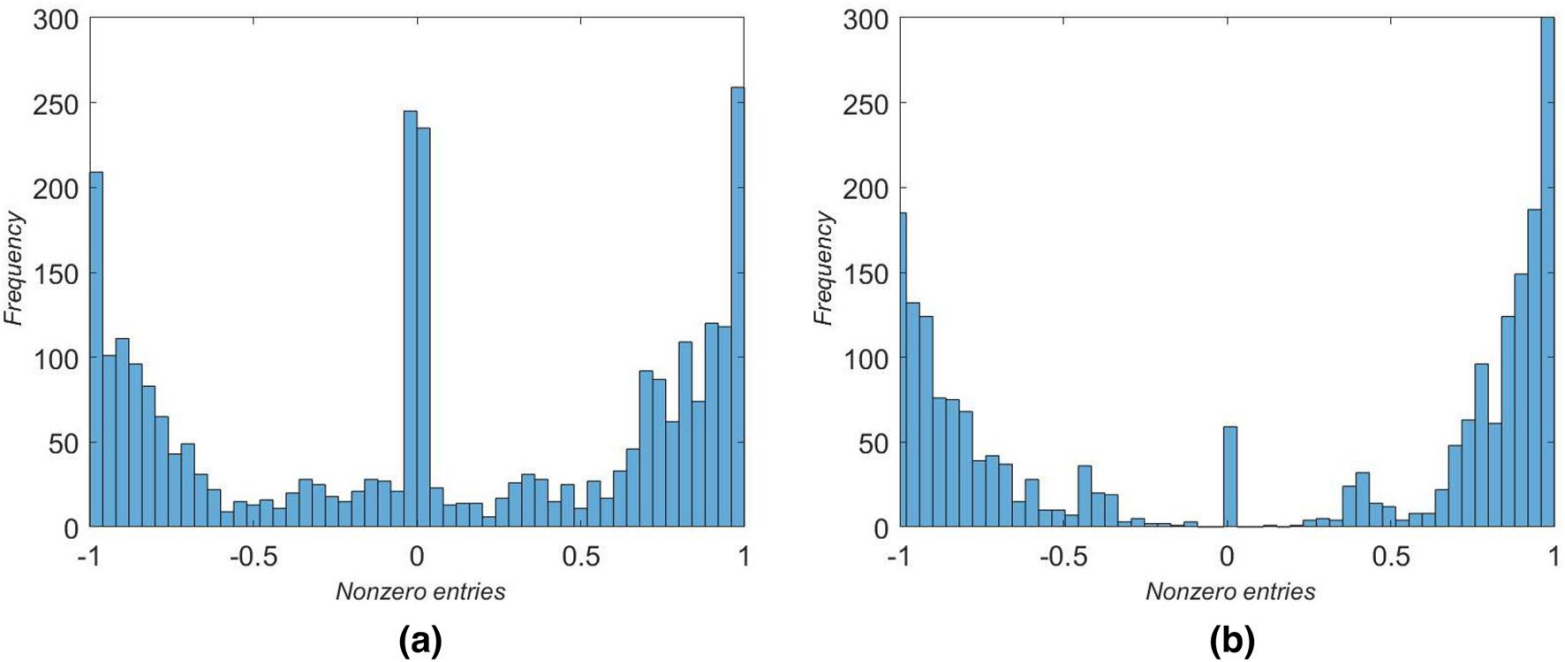
Frequency distribution of the nonzero entries in the interaction matrices derived at the country level for **a** Indonesia and **b** Bangladesh

The frequency distributions of nonzero entries in $A_\mathrm {IDN}$ and $A_\mathrm {BGD}$ are shown in Fig. 6 and are distinctly bimodal, with peaks at around $-1$ and +1. This is reflected also in the colours in Figs. 4 and 5 which contain more yellow and dark blue entries than light blue/green that would indicate values closer to zero. The distribution of entries for $A_\mathrm {BGD}$ contains a greater proportion of positive entries while the numbers of positive and negative entries for $A_\mathrm {IDN}$ are more equal. The peaks in the frequency distributions for values of the correlation coefficients may well be related to the use of the Pearson correlation coefficient for time series with significant trends (Johansen 2007; Yule 1926). In more detail, we recall the definition of the sample Pearson correlation coefficient $r_{xy}$ for pairs of observations $\{(x_1,y_1),\ldots ,(x_N,y_N)\}$ thought of as independent samples, with sample means $\bar{x}$ and $\bar{y}$, from underlying stationary distributions; $r_{xy}$ is defined by

1 $$\begin{aligned} r_{xy} = \frac{\sum _{i=1}^N (x_i-\bar{x})(y_i-\bar{y})}{\sqrt{\sum _{i=1}^N (x_i-\bar{x})^2}\sqrt{\sum _{i=1}^N (y_i-\bar{y})^2}}. \end{aligned}$$

Hence $r_{xy}$ is a measure of the strength of *linear* correlation between the time series $\{x_i\}$ and $\{y_i\}$; since it is a test of linearity the formula implies that even a small systematic increase of $\{y_i\}$ when $\{x_i\}$ is larger will return just as large a value of $r_{xy}$ as a large systematic increase. The second caveat with the use of (1) is that if both time series have a distinctive trend then these within-series effects can obscure the more subtle between-series correlation. This effect can be accommodated by applying (1) to the differences between the time series rather than the absolute values in the time series themselves. These effects would be of interest to probe further in future work, and we provide more discussion of this and related issues in “Robustness of the results” section.

Finally we note that correlations close to zero imply that the (policy) mechanism that is proposed in the framework matrix as being generally valid does not in fact provide a link for the country in question.

### Network centrality

The most natural starting point for our analysis of network structures is to consider overall statistics of the framework matrix, together with the two interaction matrices for Indonesia (IDN) and Bangladesh (BGD). Centrality measures in network science provide a quantitative measurement of the relative importance of different nodes. Of the many centrality measures available, we use eigenvector centrality since it is a well-known (Bonacich 2007) and particularly robust measure of the influence of each node in a network. It also naturally generalises to the case of networks with both negative and positive interlinkages, as we now discuss.

To begin with, consider the case in which the edge weights $A_{ij}$, which indicate the influence of target *j* on target *i*, are either positive or zero. This is the usual context for defining centrality measures, including eigenvector centrality. A larger edge weight indicates a closer association between a pair of nodes. Later in this section we will relax this constraint and allow negative edge weights.

For a directed network with non-negative edge weights, a natural (but implicit!) definition of a measure of the importance of a node is as the weighted average of the importances of the nodes that it is connected to, i.e. the importance $v_i$ of node *i* can be written as

2 $$\begin{aligned} v_i := \frac{1}{\lambda } \sum _{j=1}^n A_{ij} v_j \end{aligned}$$

where $n=169$ (in the context we consider here) is the total number of nodes in the network, i.e. the number of targets, and $\lambda$ is a parameter that allows us to rescale the sum on the right-hand side of (2). This equation at first sight appears to be entirely self-referential: the relative importance $v_i$ of node *i* is given by (up to a scale factor) the total importance of the nodes *j* that influence node *i*, weighted by the interaction strengths $A_{ij}$. One can imagine trying to find the values $v_i$ in an iterative sense, putting trial values into the terms on the right-hand side, choosing $\lambda$, and computing the values $v_i$ on the left-hand side and repeating. A more principled approach is to to rewrite the equation by multiplying up by the factor $\lambda$ to obtain the matrix-vector equation $A\mathbf{v}= \lambda \mathbf{v}$ where the vector $\mathbf{v}=(v_1,\ldots ,v_n)$ is the vector of relative importances of the nodes. This matrix-vector equation is just the mathematical definition of the eigenvalues $\lambda$ and eigenvectors $\mathbf{v}$ of the matrix *A*.

To illustrate the ideas, consider a very simple example of a three-node network in which there are directed edges from each of nodes 2 and 3 to node 1, and two directed edges connecting nodes 2 and 3, all with weight 1, i.e. $A_{12}=A_{13}=A_{32}=A_{23}=1$ so that the adjacency matrix *A* is

3 $$\begin{aligned} A= & {} \left( \begin{array}{ccc} 0 &{} 1 &{} 1 \\ 0 &{} 0 &{} 1 \\ 0 &{} 1 &{} 0 \\ \end{array} \right) \, . \end{aligned}$$

We encourage the reader unfamiliar with the notation above to draw out the three-node network and the directed edges described above, to think about how each node influences the others. The eigenvalues of this adjacency matrix *A* are 1, 0, and $-1$, and the eigenvector corresponding to the largest eigenvalue $\lambda =1$ is $\mathbf{v}=\frac{1}{\sqrt{6}}(2,1,1) \approx (0.82,0.41,0.41)$ where the factor of $1/\sqrt{6}$ is included just to make the sum of the squares of the elements of $\mathbf{v}$ equal to 1. We conclude that node 1 is the most important node in the network, and that nodes 2 and 3 are equally important.

Returning to the general case, the mathematical theory of properties of eigenvalues and eigenvectors is well-known and has been used in network science for many years to help understand properties of networks. The theory for matrices that have only non-negative entries (i.e. no ‘trade-offs’ between targets) is the simplest starting point: for matrices *A* that have no negative entries, the Perron–Frobenius theorem guarantees the existence of an eigenvector $\mathbf{v}$ (known as the ‘leading eigenvector’) that itself has no negative entries, and which has an eigenvalue $\lambda$ that is real and larger than or equal to all the other eigenvalues. The components $v_i$ of the leading eigenvector then satisfy (2) and so can be interpreted as a self-consistent solution to (2) which provides a measure of the importance, or *centrality*, of each node in the network.

For completeness, we note that the leading eigenvector $\mathbf{v}$ is defined only up to an overall scale factor; the relative differences between components are significant but the absolute values can change if a different convention is used to normalise the eigenvector (note that in (2) we could multiply all the $v_i$ by a constant and the equation would remain true). We will use the convention that $\sum _i v_i^{2}=1$ in the remainder of the paper. The existence of a leading eigenvector, and its property of having components that are either zero or positive, means that this interpretation as a centrality measure makes sense for both undirected and directed networks as long as all entries $A_{ij}$ in the interlinkage matrix are themselves either positive or zero.

The SDG interlinakge matrices that we consider here clearly contain both positive and negative entries (i.e. where both co-benefits and trade-offs exist). In this case the strict interpretation of the leading eigenvector as a ‘centrality measure’ is more difficult since the leading eigenvector also may have both positive and negative entries, and it is difficult to assign a meaning to a negative centrality score.

However, a more general interpretation is possible by considering the dynamical behaviour of the network over time. If the network structure is ‘autocatalytic’ in the sense that progress on some targets reinforces progress elsewhere in the network then positive interlinkages between targets should lead to positive effects over time. If we let $x_i(t)$ be the ‘level of progress’ towards achieving target *i*, so that $x_i=0$ means that no progress has been made, and $x_i=1$ means that the target has been achieved, then the simplest model to describe self-reinforcing effects, and therefore changes in the levels of progress $\mathbf {x}(t)$ over time, would be

4 $$\begin{aligned} x_i(t+\Delta t) = x_i(t) + \Delta t \sum _{j=1}^n A_{ij} x_j(t) \end{aligned}$$

i.e. that the change in progress on target *i* over a time interval $\Delta t$ would be proportional to the matrix-vector product of the current state of the system and the interlinkage matrix which describes how influences propagate. Although clearly highly idealised, this enables us to generalise the concept of eigenvector centrality to networks with both positive and negative interlinkages, since over long times the solutions to (4) depend almost exclusively on the leading eigenvector. The explicit solution of (4) at time $t_k=k\Delta t$ is

5 $$\begin{aligned} \mathbf {x}(t_k)= & {} \left( I + \Delta t \, A \right) ^k \mathbf {x}_0 \end{aligned}$$

which mathematically becomes dominated by the eigenvector $\mathbf{v}$ corresponding to the largest eigenvalue $\lambda$ when *k* becomes large. So the components $v_i$ of the leading eigenvector $\mathbf{v}$ can be interpreted as the rates at which the ‘levels of progress’ $v_i$ either increase or decrease over time. If all components $v_i$ are positive and $\lambda$ is larger than any other eigenvalue of *A* then the matrix *A* was said to be *self-consistent’* in Dawes (2020) where this idea is explored in detail for a different interlinkage network. We may therefore interpret negative components in the leading eigenvector as targets for which progress might actually decrease over time due to trade-offs within the network.

In summary, having computed the network interlinkage matrix *A*, the eigencentrality computation is just the computation of the leading (i.e. largest positive) eigenvalue of *A* and the corresponding leading eigenvector. The form of the leading eigenvector is typically a very good guide to the intrinsic response of the network in the sense that it describes how progress on the SDGs reinforces itself over time, due to the interlinkages coded in the network structure.

### Network hierarchy

The design of network statistics to capture directionality is a topic of significant current interest across many areas of application in network science. Network analyses of ecosystems have for many years aimed to compute and understand food webs describing predator–prey relationships between species. Such food webs are naturally layered through the directed relationships between predator–prey pairs. In the food web literature such layers are referred to as ‘trophic layers’. More generally, this motivates the natural question of whether a given directed network can be organised into a collection of similar ‘trophic layers’, so that the directed edges in the network connect species in adjacent layers, with all the edges pointing in the same direction. This question was recently explored by MacKay et al. (2020) and we follow their presentation in this section, generalising their approach and applying it to these SDG interlinkage networks.

Mathematically, splitting a network into ‘trophic layers’ corresponds to the calculation of a ‘layer height’ value $h_i$ for each node *i*. Nodes with higher values of $h_i$ are then further ‘downstream’ in the network; a node *i* with a lower value of $h_i$ is further ‘upstream’ and therefore, in the SDG network context, could be considered to be a driver of progress on other targets downstream of it. The separation of nodes into layers can also be thought of as finding an arrangement of the network nodes in which as many directed edges as possible can be arranged to point in the same direction as each other (i.e. upwards in this setup). In the remainder of this section we translate this intuitive concept of network hierarchy into a mathematical formulation that allows us to compute it quantitatively for a specific network.

A simple mechanism to describe the separation of network nodes into layers is given by minimisation over $\mathbf {h}$ of the function

6 $$\begin{aligned} F(\mathbf {h}; A):= & {} \frac{1}{\sum _{i,j=1}^n |A_{ij}|} \sum _{i,j=1}^n |A_{ij}| (h_i-h_j-1)^2 \end{aligned}$$

which is a weighted sum of the height values $\mathbf {h}=(h_1,\ldots ,h_n)$ where $n=169$ is the total number of SDG targets, *A* is the interlinkage network, and $h_i$ is the layer height of node *i*. The theory presented by MacKay et al. (2020) assumes that the network has no negatively weighted edges, so we replace the interlinkage strength $A_{ij}$ by its absolute value $|A_{ij}|$. The form of (6) indicates that $F(\mathbf {h})$ will be minimised by choices of the $h_i$ that put a node *i* on a level (assumed to be spaced out roughly by the integers) below a node *j*, so that $h_i - h_j \approx 1$, when there is a directed edge $j \rightarrow i$. An explicit equation for the levels $\mathbf {h}$ that minimise $F(\mathbf {h})$ can be deduced by differentiating (6) with respect to $h_i$ and setting $\partial F / \partial h_i=0$ for all *i*. This results in a linear equation which can be straightforwardly solved for the vector $\mathbf {h}$:

7 $$\begin{aligned} \Lambda \mathbf {h}= & {} \mathbf {k}^{in} - \mathbf {k}^{out} \end{aligned}$$

where $\mathbf {k}^{in}_i:=\sum _j A_{ij}$ is the in-degree of node *i*, $\mathbf {k}^{out}_j:=\sum _i A_{ij}$ is the out-degree of node *j*, and the Laplacian matrix $\Lambda :=\mathrm {diag}(\mathbf {k}^{in} + \mathbf {k}^{out}) - A - A^T$ where $\mathrm {diag}(\mathbf {u})$ is the $n \times n$ matrix formed by putting the entries of the vector $\mathbf {u}$ on the diagonal and zeros elsewhere.

While the function $F(\mathbf {h})$ defined in (6) captures the desired sense of hierarchy in possibly the simplest form, it is mathematically slightly unsatisfactory in two ways. Firstly, for a weighted network it is more natural that the preferred spacing $h_i-h_j$ correspond to the mean nonzero edge weight $\langle |A|\rangle$ rather than be fixed at 1. It is also potentially of interest to examine changing the weighting of each term to include a different power of $|A_{ij}|$ since in different situations a high value of an edge weight could indicate that nodes should be placed at very similar levels in the hierarchy rather than be pushed apart to separate levels. Hence a generalised version of (6) introducing the mean edge weight $\langle |A|\rangle$ and an exponent $\alpha$ into the weighting term would be

8 $$\begin{aligned}&{\tilde{F}}_\alpha (\mathbf {h};\,A^{\odot \alpha }) = \frac{1}{\sum _{i,j=1}^n |A_{ij}|^{\alpha }} \nonumber \\&\quad \sum _{i,j=1}^n |A_{ij}|^\alpha \left( h_i - h_j - \langle |A|\rangle \right) ^2 . \end{aligned}$$

where the notation $A^{\odot \alpha }$ indicates that the power $\alpha$ is applied elementwise to the matrix (i.e. to each matrix entry individually), and only to nonzero entries; for $\alpha <0$ we preserve the zeros in the matrix as zeros.

A straightforward rearrangement of (8) shows that this generalised version can be written in terms of the original function *F* as follows:

9 $$\begin{aligned}&{\tilde{F}}_\alpha (\mathbf {h};\, A^{\odot \alpha }) = \frac{\langle |A|\rangle ^2}{\sum _{i,j=1}^n |A_{ij}|^{\alpha }} \nonumber \\&\quad \sum _{i,j=1}^n |A_{ij}|^\alpha \left( \frac{h_i}{\langle |A|\rangle } - \frac{h_j}{\langle |A|\rangle } - 1 \right) ^2 \nonumber \\&\quad = \langle |A|\rangle ^2 F({\tilde{\mathbf {h}}}; A^{\odot \alpha }), \end{aligned}$$

where ${\tilde{\mathbf {h}}}:=\mathbf {h}/ \langle |A|\rangle$ is a rescaled version of the layer heights $\mathbf {h}$. Hence we see that minimising $F_\alpha$ over $\mathbf {h}$ is equivalent to minimising *F* over ${\tilde{\mathbf {h}}}$ when we replace the adjacency matrix *A* by the matrix $A^{\odot \alpha }$ defined above. In consequence, Eq. (9) shows that the relative ordering of the layer heights at the minimum is not affected by the mean edge weight.

We turn now to the role of the exponent $\alpha$ and the use of the scaled edges given by the matrix $A^{\odot \alpha }$; this is more complicated to determine. The most straightforward special case to consider is the one in which all nonzero entries in *A* are equal: then the matrix $A^{\odot \alpha }$ is just a scalar multiple of *A* and so the relative layer heights that minimise $F\alpha$ do not depend on $\alpha$. For positive values of $\alpha$ interlinkages that have large positive values will tend to push nodes further apart from each other: high value links will cause nodes that it connects to become strongly separated. Conversely, when $\alpha$ is negative high value links will result in close connections between nodes. This is aligned with the philosophy behind the seven point scale $-3,\ldots ,0,\ldots ,+3$ proposed by Nilsson et al. (2016) where an interaction score of $+3$ corresponds to an ‘indivisable’ influence of one target on another; in their words ‘[the achievement of one target] is inextricably linked to the achievement of another [target].’

A significant shift, relative to others, in the position of a target or a Goal as $\alpha$ varies indicates that such a target or Goal is influenced by connections of varying strengths. This is because for large positive $\alpha$ the linkages (edges) with large values tend to push connected Goals further apart from each other; hence the large positive $\alpha$ regime could be termed the ‘outcome space’ since positive $\alpha$ weights stronger links with higher values, in accordance with the equal effort differential equation ${\dot{\mathbf {x}}}=A\mathbf {x}+ \mathbf {m}$ considered by Dawes (2020). Negative $\alpha$ could be thought of as the ‘policy space’ since here strongly connected targets or Goals move closer together and so potentially could be thought of as able to be addressed through related policy actions.

A final, and slightly technical, point is that the minimisation can only assign a layer value $h_i$ to all nodes *i* when the network is ‘weakly connected’ (i.e. when the network would contain a path connecting every pair of nodes if all edges were considered to be undirected). To ensure this connectedness for the Bangladesh and Indonesia networks we use modified versions ${\tilde{A}}_\mathrm {BGD}$ and ${\tilde{A}}_\mathrm {IDN}$ of the interlinkage matrix defined as follows:

10 $$\begin{aligned} {\tilde{A}}_{\mathrm {BGD}\, ij} = {\left\{ \begin{array}{ll} A_{\mathrm {BGD} \, ij} &{} \quad \text {if} \ A_{\mathrm {BGD} \, ij} \ne 0 \\ \epsilon &{} \quad \text {if} \ A_{\mathrm {BGD} \, ij} = 0 \ \text {but} \ A_{ij} = 1 \\ 0 &{} \quad \text {if} \ A_{\mathrm {BGD} \, ij} = A_{ij} = 0 \, , \end{array}\right. } \end{aligned}$$

where *A* is the framework matrix and $A_\mathrm {BGD}$ is the unmodified interlinkage matrix for Bangladesh. Similarly, for the modified Indonesia interaction matrix we define

11 $$\begin{aligned} {\tilde{A}}_{\mathrm {IDN}\, ij} = {\left\{ \begin{array}{ll} A_{\mathrm {IDN} \, ij} &{} \quad \text {if} \ A_{\mathrm {IDN} \, ij} \ne 0 \\ \epsilon &{} \quad \text {if} \ A_{\mathrm {IDN} \, ij} = 0 \ \text {but} \ A_{ij} = 1 \\ 0 &{} \quad \text {if} \ A_{\mathrm {IDN} \, ij} = A_{ij} = 0 \, . \end{array}\right. } \end{aligned}$$

In both cases we set $\epsilon =10^{-8}$. The reason for requiring the presence of these low value interlinkages is the unfortunate lack of data availability for the computation of all the edges that the expert analysis suggests should exist. Numerical tests confirm that the results we will present in “Results” section are independent of the choice of $\epsilon$ when it takes values this small. The levels $h_i$ remain determined by the interlinkages in each country-specific interaction matrix, but the presence of the $\epsilon$-weighted edges serves to connect the network into a single component, allowing all relative levels to be uniquely determined.

To summarise, once the network interaction matrix *A* has been computed, the network hierarchy can be deduced just by solving (7) for the levels $h_i$ for each network node *i*. This collection of levels $h_i$ are the components of the vector $\mathbf {h}$ that minimises the ‘trophic confusion’ quantity $F(\mathbf {h},A)$ defined in (6). The remainder of this subsection discussed variations and extensions of this basic setup.

## Results

### Network interactions

#### Framework matrix

We start by looking at the framework matrix *A* determined by IGES. Since *A* has only positive and zero entries, its leading eigenvector is guaranteed to have both the centrality and the ‘autocatalytic’ interpretations described above. The pattern of non-zero entries in *A* is illustrated in Fig. 2.

Out of a possible 169$\times$168=28,392 nonzero entries (rather than 169$\times$169 since there are no self-interactions), *A* has 8759 that are identified as possible interlinkages between targets, around $31\%$ of the maximum possible. Thus the framework matrix *A* provides a considerable constraint on the possible networks of interactions at country levels. The in-degree and out-degree of network nodes varies significantly. Targets 3.a and 17.4 have the lowest in-degree. Target 3.a is influenced (potentially) by at most 10 other targets. This makes sense in view of its very specific formulation: ‘Strengthen the implementation of the WHO Framework Convention on Tobacco Control in all countries, as appropriate.’ Target 17.4 is concerned with debt financing and debt relief which again is a specific issue hardly referred to elsewhere, leading to a small number of other nodes influencing it directly. In both cases it is clear that these targets are not well supported by progress on other targets within the network of interlinkages, and as a result specific policy actions are highly likely to be required to ensure that these targets are met.

In contrast, target 2.3 (‘double agricultural productivity’) has the highest in-degree, being influenced by 114 other targets. In terms of out-degrees, targets 2.c (‘ensure the proper functioning of food commodity markets... to limit price volatility’) and 4.2 (early childhood development) have the lowest score, influencing only 9 other targets, while targets 17.9 (‘enhance capacity building’) and 17.18 (‘increase the availability of disaggregated data’) each influence all other 168 targets. These observations are in line with the eigenvector centrality results presented by Zhou and Moinuddin (2017) who carried out a comparison of the results of several different centrality measures on the framework matrix; we recall also here the discussion in “Network centrality” section of the meaning of eigencentrality as a measure of the relative importance of each node in the network, building on the notions of in-degree and out-degree.

**Fig. 7:**
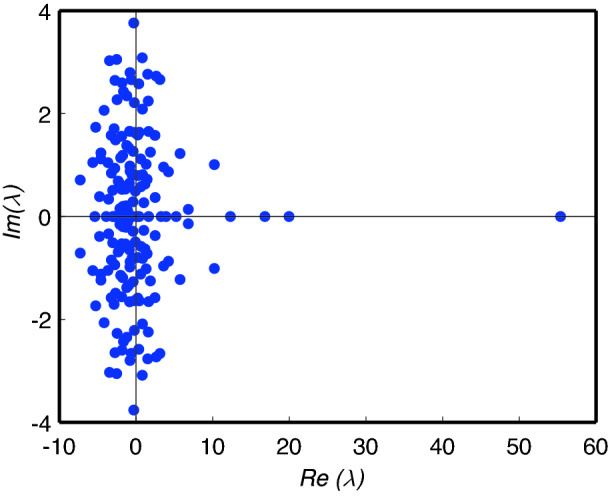
Eigenvalues $\lambda$ of the framework matrix *A* in the complex plane, i.e. where the horizontal axis is the real part $Re(\lambda )$ and the vertical axis is the imaginary part $Im(\lambda )$. Note the single real eigenvalue $\lambda =55.364$ that appears far to the right of the other eigenvalues

Computation of the eigenvalues and eigenvectors of *A* reveals that the largest eigenvalue $\lambda = 55.364$ (to 3 decimal places) lies a long way to the right of the remainder of the cluster of eigenvalues around the origin, see Fig. 7. This indicates that the framework matrix has a dominant structural ‘mode of response’ which organises the way that influences between targets propagate through the network. As discussed previously in “Network centrality” section, the behaviour of solutions (5) to the discretised equation (4) describing self-reinforcing growth is dominated by the eigenvector corresponding to $\lambda$.

**Fig. 8:**
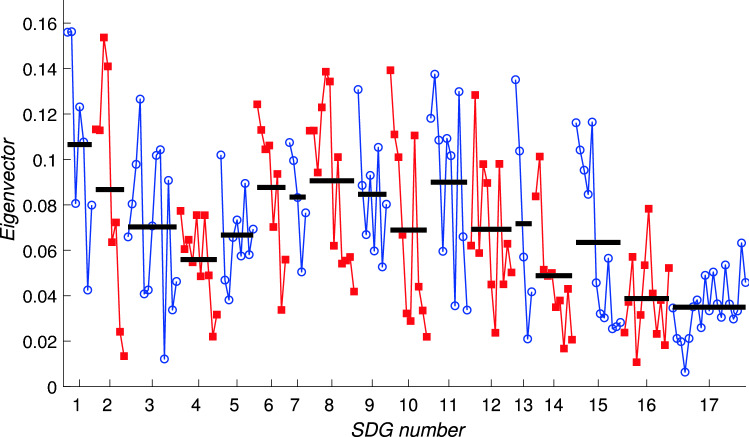
The components of the leading eigenvector for the framework matrix *A*, plotted component by component. This gives the ‘generalised centrality’ score for each target. For clarity, targets corresponding to a single Goal are joined by solid lines with the same symbol and colouring; blue lines joining open circles indicate odd-numbered SDGs while red lines joining square symbols indicate even-numbered SDGs. Horizontal black bars indicate the average of the components for each SDG

To examine this intrinsic behaviour we plot in Fig. 8 all 169 components of the leading eigenvector. These are equivalently the eigencentralities of nodes in the network of targets. Targets within odd-numbered SDGs are coloured blue and are shown by the blue circles; targets within even-numbered SDGs are coloured red and use the red squares, in order to distinguish them more clearly. The horizontal black bars indicate the averages for each Goal, taken over the relevant set of targets. There is clearly a high degree of variability across the targets, and within each SDG, which indicates that the relative importances of targets within a goal can vary significantly. Therefore an analysis at the level of individual targets is warranted—the averages across all targets within each goal do not reveal this target-level variation. There are several specific points of interest to note.

- First, no component is zero which implies that every target is influenced by some other target; the network is connected.
- Second, within almost every Goal there is a trend that the components corresponding to the first few targets take higher values than those corresponding to later targets, especially the ‘means of implementation’ targets. This indicates that the ‘means of implementation’ targets are systematically less well supported by the network as a whole compared to the outcome-related targets. This is likely to reflect imbalances in the literature sources; the targets corresponding to direct outcomes are discussed more frequently in the SDG literature than the means of implementation targets.
- Third, the broad pattern of levels of components across the different SDGs is very similar to that observed in other datasets at the whole-Goal level (Dawes 2020, 2021). Goals 1, 2 and 3 are higher than Goals 4 and 5; later Goals, particularly SDGs 14, 16 and 17, are much less well supported by the network interactions.

This last point is closely related to the general pattern of non-zero entries in *A*. As in previous analyses at the Goal level, the framework matrix has fewer non-zero entries in its lower left corner than across the top of the matrix. This asymmetry shows that SDGs with lower numbers, in particular SDGs 1, 2 and 3, do not themselves drive progress on targets in other Goals to the same extent that targets elsewhere contribute to targets in Goals 1, 2 and 3.

#### Country-level matrices

**Fig. 9:**
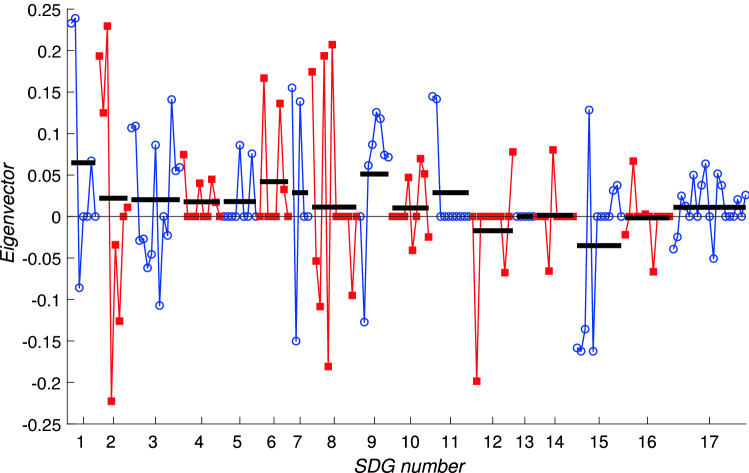
The components of the leading eigenvector for the interaction matrix for Indonesia, $A_\mathrm {IDN}$, plotting all 169 components (horizontal axis) against the eigenvector centrality measure (vertical axis). For clarity, targets corresponding to a single Goal are joined by solid lines with the same symbol and colouring; blue lines joining open circles indicate odd-numbered SDGs while red lines joining square symbols indicate even-numbered SDGs. Horizontal black bars indicate the average of the components for each SDG. Note that there are many individual components of the eigenvector that are zero

Figure 9 shows the leading eigenvector for the Indonesia data. The interlinkage matrix now contains significantly fewer nonzero entries (only 2824) due to the data gaps. Of these, 1502 are positive and 1322 are negative. The sparsity of the interlinkage matrix means that many components of the leading eigenvector are zero, and the large number of negative entries makes the interpretation of the components of the leading eigenvector as a centrality measure problematic. However, the interpretation as a progress measure is still valid. The averages across each Goal indicated by the black bars in Fig. 9 follow a similar pattern to those in Fig. 8 with Goals 1, 6, and 9 scoring most highly. Key concerns include Goals 12 and 15 which have several negative components showing that the negative influences from other Goals may serve actually to reverse progress on targets within these Goals.

**Fig. 10:**
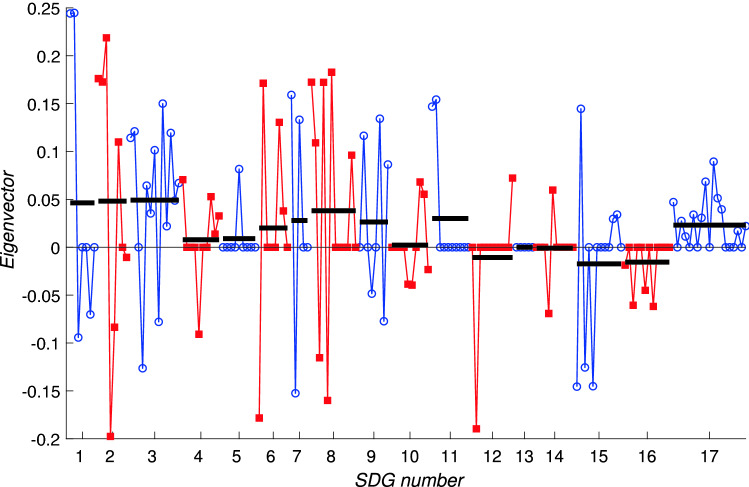
The components of the leading eigenvector for the interaction matrix for Bangladesh, $A_\mathrm {BGD}$, plotting all 169 components (horizontal axis) against the eigenvector centrality measure (vertical axis). For clarity, targets corresponding to a single Goal are joined by solid lines with the same symbol and colouring; blue lines joining open circles indicate odd-numbered SDGs while red lines joining square symbols indicate even-numbered SDGs. Horizontal black bars indicate the average of the components for each SDG. Note that there are many individual components of the eigenvector that are zero

Turning to the data for Bangladesh we note that the data has similar overall statistics: within the interaction matrix $A_\mathrm {BGD}$ there are 2186 nonzero entries, even fewer than for $A_\mathrm {IDN}$, and of these 1218 are positive while 968 are negative. As with the Indonesia data, many components of the leading eigenvector, shown in Fig. 10 are zero, showing that the network has become disconnected. The leading eigenvector for the Bangladesh data is shown in Fig. 10 which shares many similar features with that for the Indonesia data shown in Fig. 9. As time evolves, due to self-reinforcing effects within the network we therefore expect that for Bangladesh, more progress will be made on Goals 1, 2, 3 and 8, while much less, and perhaps even negative progress, on average, will occur for targets within Goals 12, 15 and 16.

At the target level, these results would suggest that for both countries there is the possibility of stagnation, or indeed negative progress over time, on a significant number of targets. Direct comparisons between the two countries are however complicated, not least due to the very large number of zero components in Figs. 9 and 10 (around half in each case: 85 in the case of Indonesia and 89 for Bangladesh). These are due to the unavailability of data, leading to zeros in the interlinkage matrices. The numbers of nonzero components of the leading eigenvectors are extremely similar: 54 positive components and around 30 negative components; the uncertainties indicated by the number of zero components shows that no conclusions should be drawn from precise numbers of positive or negative components concerning whether or not one country is closer to achieving the SDGs or not.

Table 2 compares the targets with the most positive eigenvector components for the framework matrix and the two country-specific matrices, i.e. the ones that rank as most supported by the network in this study. In each case these are also compared with the ranking of targets reported in the IGES Research Report (Zhou and Moinuddin 2017) where targets are rank-ordered by their in-degree. The positions of these targets in the relevant tables in Zhou and Moinuddin (2017) is given in the columns headed ‘ZM-2017’, with ‘(–)’ indicating that the target does not appear in the top 20 for those tables. It is clear that there is substantial correlation between the leading eigenvectors of *A*, $A_\mathrm {IDN}$ and $A_\mathrm {BGD}$ with some interesting exceptions, for example target 8.1 which appears much higher on the list for $A_\mathrm {IDN}$ and $A_\mathrm {BGD}$ than for the framework matrix. A straightforward comparison with Zhou and Moinuddin (2017) suggests a level of agreement that is significantly better than random, despite the differences in methodology, as we now explain. If the two analyses resulted in completely random selections of a ‘top 20’ from the 169 targets then we would expect on average two or three targets to appear in the same lists by chance. In fact, the framework matrix calculations and Indonesia matrices have five in common, while the Bangladesh calculation has nine of the same top twenty out of 169. This therefore indicates a much higher degree of agreement than would be expected by chance, and a level of robustness between these two calculations. Overall, the lists of most positive components are heavily skewed towards the first three SDGs, with seven out of the top twenty coming from SDGs 1–3 in the framework matrix and eight out of the top twenty in each of the country-specific cases. This illustrates again the extent to which we should anticipate that more progress will be made on SDGs 1–3 compared to the remaining Goals.

**Table 2:** Most highly ranked targets, i.e. those on which the analysis predicts most positive progress looking at the leading eigenvectors for the framework matrix *A* and the country-specific matrices $A_\mathrm {IDN}$ and $A_\mathrm {BGD}$

	Top 20 most promising targets					
	*A*—Framework		$A_\mathrm {IDN}$—Indonesia		$A_\mathrm {BGD}$—Bangladesh	
Rank	Target	ZM-2017 (*)	Target	ZM-2017 (**)	Target	ZM-2017 (**)
1	1.2	(–)	1.2	(–)	1.2	(7)
2	1.1	(–)	1.1	(–)	1.1	(8)
3	2.3	(2)	2.3	(3)	2.3	(1)
4	2.4	(11)	8.6	(16)	8.6	(–)
5	10.1	(–)	8.4	(–)	2.1	(–)
6	8.5	(9)	2.1	(–)	2.2	(–)
7	11.2	(–)	8.1	(–)	8.1	(–)
8	13.1	(–)	6.2	(–)	8.4	(18)
9	8.6	(–)	7.1	(15)	6.2	(4)
10	9.1	(12)	11.1	(–)	7.1	(9)
11	11.a	(–)	11.2	(–)	11.2	(20)
12	12.2	(–)	3.b	(–)	3.9	(–)
13	3.4	(–)	7.3	(–)	11.1	(11)
14	6.1	(3)	6.6	(7)	15.2	(–)
15	1.4	(–)	15.4	(–)	9.a	(–)
16	8.4	(–)	9.5	(–)	7.3	(–)
17	11.1	(–)	2.2	(–)	6.6	(3)
18	15.5	(–)	9.a	(18)	3.2	(–)
19	15.1	(–)	3.2	(–)	3.b	(–)
20	2.1	(–)	3.1	(–)	9.2	(–)

The numbers in parentheses $(*)$ in the columns headed ‘ZM-2017’ give the position of those targets in Table 10 (*) and the relevant parts of Table 11 (**) in Zhou and Moinuddin (2017); in that study the targets are ranked by in-degree for the framework matrix and for each country

**Table 3:** Targets for which the components of the leading eigenvectors for $A_\mathrm {IDN}$ and $A_\mathrm {BGD}$ are negative, implying that significant trade-offs exist with other targets in the network

SDG	Targets at risk			
	$A_\mathrm {IDN}$—Indonesia		$A_\mathrm {BGD}$—Bangladesh	
	#	Targets	#	Targets
1	1	1.3	2	1.3, 1.a
2	3	2.4, 2.5, 2.a	3	2.4, 2.5, 2.c
3	6	3.3 - 3.6, 3.8, 3.a	2	3.4, 3.8
4	0		1	4.5
5	0		0	
6	0		1	6.1
7	1	7.2	1	7.2
8	4	8.2, 8.3, 8.5, 8.a	2	8.3, 8.5
9	1	9.2	2	9.4, 9.b
10	2	10.6, 10.c	3	10.5, 10.6, 10.c
11	0		0	
12	2	12.2, 12.a	1	12.2
13	0		0	
14	1	14.4	1	14.4
15	4	15.1, 15.2, 15.3, 15.5	3	15.1, 15.3, 15.5
16	2	16.1, 16.8	4	16.1, 16.3, 16.6, 16.8
17	3	17.1, 17.2, 17.11	0	

For each SDG, the number (indicated by ‘#’) and then a list of the specific targets, is given. Data for Indonesia and for Bangladesh is listed in separate columns

Although the positive values of correlation coefficients point result, for both Indonesia and Bangladesh, from positive trends in the indicator time series, it is important to note that just because the trend is positive this does not imply that the country is on track to achieve the target by 2030. Indeed as ESCAP (2020) reports, most countries in the Asia-Pacific region are not on track to achieve most of the targets by 2030. However, these results do indicate that steady progress, and self-reinforcing effects between targets, generate positive progress on at least this subset of the targets. As the COVID-19 pandemic from 2020 onwards has shown, there are also unexpected external influences that may disrupt (or potentially accelerate) progress so the idea of steady progress year on year until 2030 is unlikely to be realistic.

The results for the framework matrix serve to illustrate that the set of connections itself, and the structure that it imposes on the network, is also related to an idea of prioritisation within the set of targets. The framework matrix does not correspond to any real country but it serves to indicate that some targets (those listed in Table 2) play a more central role in the network.

Table 3 summarises, goal by goal and for Indonesia and Bangladesh separately, the targets for which the components of the leading eigenvector are most negative. This indicates that the aggregate network influence on these targets comprises more trade-offs than co-benefits, and that progress on other targets could come at the expense of progress on these targets. Rather than speak of ‘negative progress’ on these targets it may well be that there is in practice only stagnation, but, for the environmental targets in SDGs 14 and 15 that appear to be at risk, further deterioration is of course possible. Alternatively, and not accounted for in this analysis, these targets may require specific resource allocations to alleviate these trade-offs. There is a high degree of overlap between the results for the two interlinkage matrices, with 17 targets appearing in both lists, and only a further 22 appearing for either one country or the other; $44\%$ are common to both countries. There appear to be particular difficulties apparent in meeting targets in Goals 2, 3, 8, 15 and 16. Overall we note that the fluctuations between different components of the leading eigenvector for targets within one Goal can be very large. This indicates that results based on analysis at the whole SDG level may well obscure issues that relate to individual targets and so a target-level analysis has considerable value over aggregated Goal-level analyses.

Looking at each country separately, this analysis exposes the particular challenges that Indonesia and Bangladesh appear to face. We note that it is not so straightforward to make comparisons directly between the two countries, since the interlinkage network does not describe the absolute level of progress on each target. In terms of interlinkages, for Indonesia we can see that progress on targets within SDGs 3, 8, 15 and 17, generally speaking, are supported less well than targets within other SDGs. Similarly, for Bangladesh there are clusters of targets on which progress is less well reinforced within SDGs 10, 15 and 16. A number of targets are also potentially less well supported systematically for both countries; this is due to the similarities in the interlinkage matrices; we recall that both country-specific matrices are constrained by the framework matrix described in the IGES methodology in “Data” section and Fig. 2.

### Network hierarchy

**Fig. 11:**
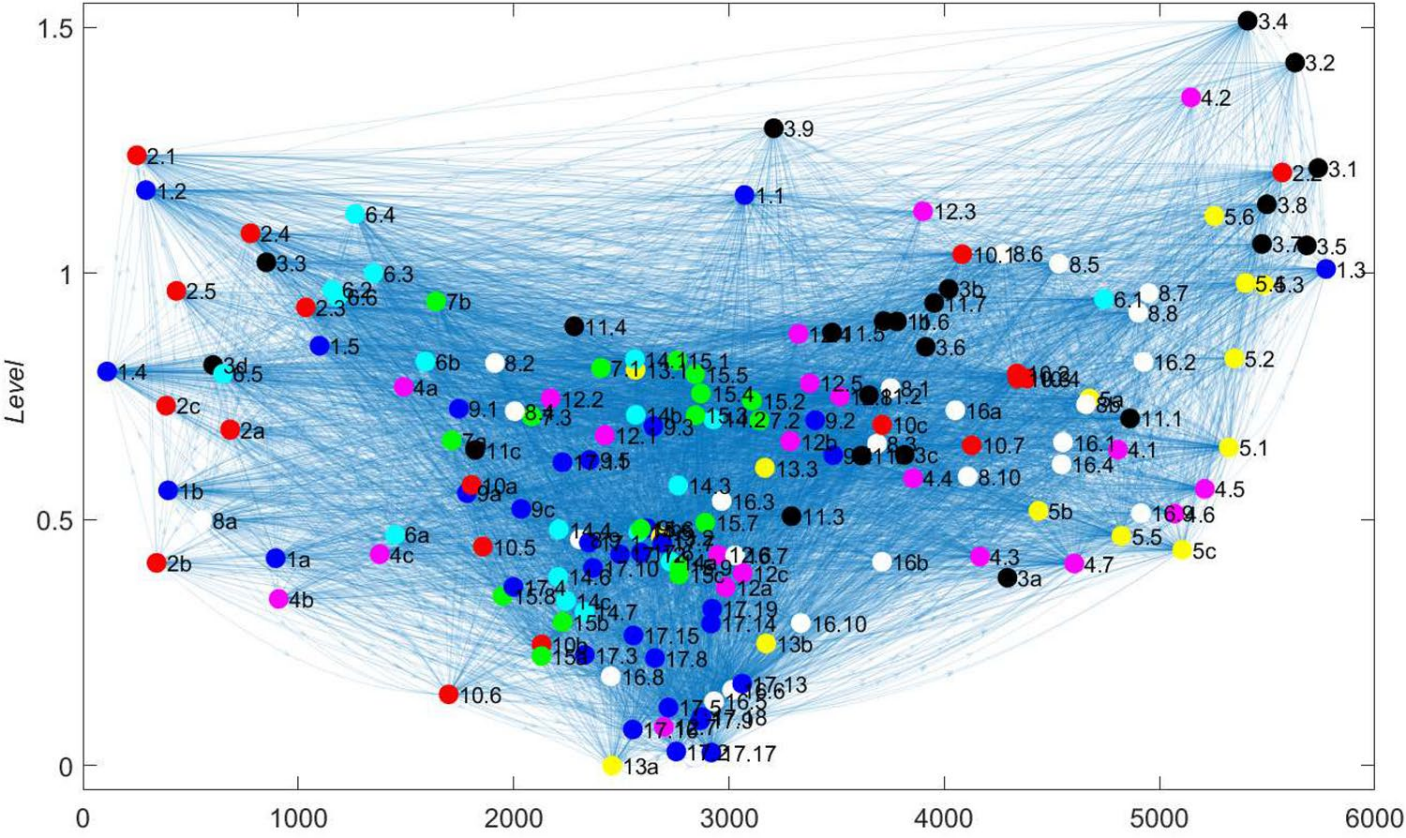
Representation of the framework network *A* using the levels $h_i$ for each of the 169 targets that minimise the function $F(\mathbf {h})$ defined in (6). The vertical axis indicates the value of $h_i$; the horizontal axis serves just to organise the network for visualisation purposes. Targets corresponding to the same SDG are coloured the same colour; the same colour is used for at most three SDGs

**Fig. 12:**
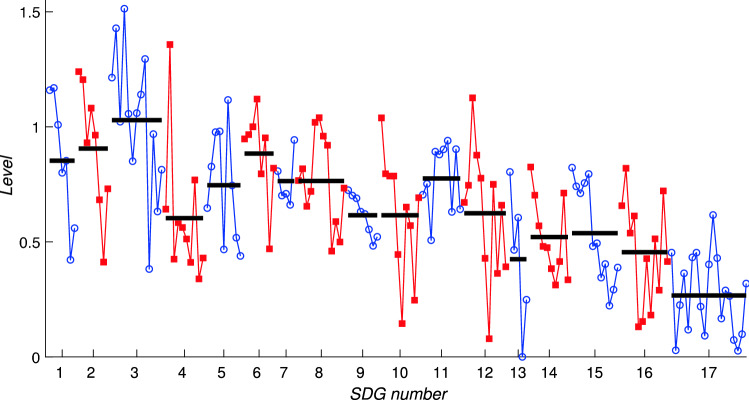
Levels $h_i$ for each of the 169 targets that minimise the function $F(\mathbf {h})$ defined in (6), for the framework network *A*. The vertical axis indicates the value of $h_i$ and is equal to the vertical positions of the dots in Fig. 11. Horizontal black bars indicate the average level of the targets within each SDG

In this section we comment on the results obtained by computing the levels $\mathbf {h}$ that result from minimising the function $F_\alpha$ defined in (9). To streamline the discussion we focus mainly on showing results for the cases $\alpha =1$ and $\alpha =-1$ in this section. Additional figures illustrating how the results vary for $\alpha$ in the range $-1,\ldots ,1$ are shown in the Electronic Supplementary Material. For all these figures, it is important to emphasise that the levels $h_i$ are always a relative measure of importance; the calculations are unaffected by an absolute shift in the values $h_i$ since only the differences $h_i-h_j$ are present in Eqs. (6) and (9). In Figs. 11 and 12 we resolve this additional degree of freedom by setting the lowest level to take the value zero.

We look first at the framework matrix *A*: Figs. 11 and 12 present the levels $\mathbf {h}$ that minimise the trophic hierarchy function $F(\mathbf {h};A)$ defined in (6), using the framework matrix *A* as a base case. The vertical position of target *i* indicates the level $h_i$ and the horizontal position is constructed so as to present the network in a manner that allows edges to be drawn as close to straight lines as possible. This results in some ‘clustering’ of nodes that have many common neighbours but it is not a formal clustering algorithm. The overall directionality of the network is from lower levels to higher ones: arrows in general point upwards in the figure.

The variation in the level of targets within each SDG is clear, for example for SDG 5 (yellow dots on the right hand side of the figure) the levels of the individual targets range from approximately 0.4 to around 1.2. Targets 3.4, 3.2, 4.2, 3.9, and 2.1 emerge at the highest levels, indicating that these targets are at the heads of many arrows and are influenced by many other targets that lie ‘upstream’ of them in the directed network.

**Fig. 13:**
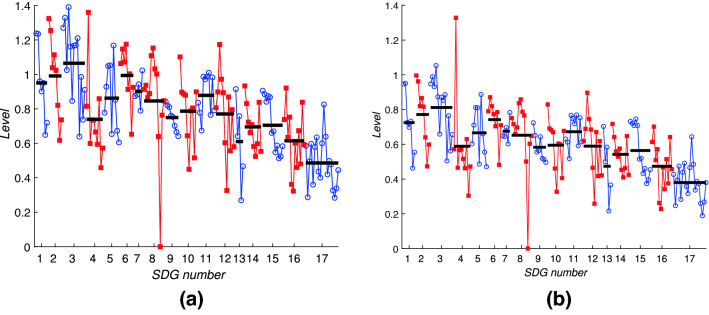
Levels $h_i$ for each target $i=1,l\dots ,169$, together with averages for each SDG (black horizontal lines) computed for $\alpha =1$. **a** Bangladesh matrix ${\tilde{A}}_\mathrm {BGD}$; **b** Indonesia matrix ${\tilde{A}}_\mathrm {IDN}$. Each matrix is regularised by inserting the value $\epsilon =10^{-8}$ for those entries where no value is given in the IGES Toolkit but the framework matrix suggests an interlinkage is possible

Figure 12 then plots the same levels for each target in order, omitting the network connections shown in Fig. 11, but bringing out the variation with the ordering of the SDGs. The vertical positions of points inF Figs. 11 and 12 are equal. Averaged over each SDG we see that the levels for Goals 4 and 13–17 are lower than the remaining Goals, suggesting that on average these SDGs lie upstream of the others in the network. At the level of individual targets, targets 13.a, 17.2 and 17.17 lie furthest upstream, indicating that these are least influenced by other targets compared to the level of influence they have on others. In terms of sense-checking the methodology, it is perhaps reassuring to see that all targets within Goal 17 are assigned a low level in the hierarchy, indicating that they lie upstream of many other targets within the 2030 Agenda and therefore have substantial influence (Fig. 13).

We remark that this calculation, because it takes account of the entire network structure, differs from the just computing the out-degree of each node: as noted in “Data” section, targets 17.9 and 17.18 would on that measure be considered the most influential since they have the largest out-degree possible for the network, being connected to all other nodes.

At a country-specific level we can apply the hierarchy algorithm to the modified adjacency matrices for Bangladesh ${\tilde{A}}_\mathrm {BGD}$ and Indonesia ${\tilde{A}}_\mathrm {IDN}$ defined in (10) and (11) respectively. In both cases target 8.10 ‘Strengthen the capacity of domestic financial institutions to encourage and expand access to banking, insurance and financial services for all.’ emerges as the target that lies furthest upstream, implying that target 8.10 has a greater influence on other targets than any other target has on it.

A small number of other targets also have particularly low levels, often noticeably lower than the levels for other targets belonging to the same SDG. This set is extremely similar for the two countries, and indeed for the framework matrix, and includes the following:

- 4.b—Expand scholarships for developing countries *
- 10.6—Inclusion of developing countries in global decision making
- 12.7—Promote public green procurement
- 13.a—Finance developing countries for mitigation *
- 17.2—Implement ODA commitments *
- 17.17—Promote multistakeholder partnerships.

This list therefore comprises the actions that in network terms are the most fundamental and are least likely to follow from others. It is of interest to note that three of these (indicated by the *) are phrased within the SDGs as being at least in part the responsibility of developed country partners, as compared to actions that are internal to the developing country itself.

As an aid to interpretation we note that the level $h_i$ for a particular target is related to the difference between its in-degree and its out-degree, as (7) shows since this difference is precisely the right hand of (7). However, the (Laplacian) matrix $\Lambda$ then plays the role of adjusting the levels to minimise the function *F* and so find the set of relative levels that best describes the hierarchy in the network. Therefore the calculation overall amounts to a modified version of this analysis of the difference between in-degree and out-degree, in effect taking into account those differences for neighbouring nodes and treating the network as a whole. The underlying philosophy of constructing a self-consistent set of levels $h_i$ for the whole network is very similar to the construction of a self-consistent set of centrality measures, as discussed in “Network centrality” section for eigenvector centrality.

**Fig. 14:**
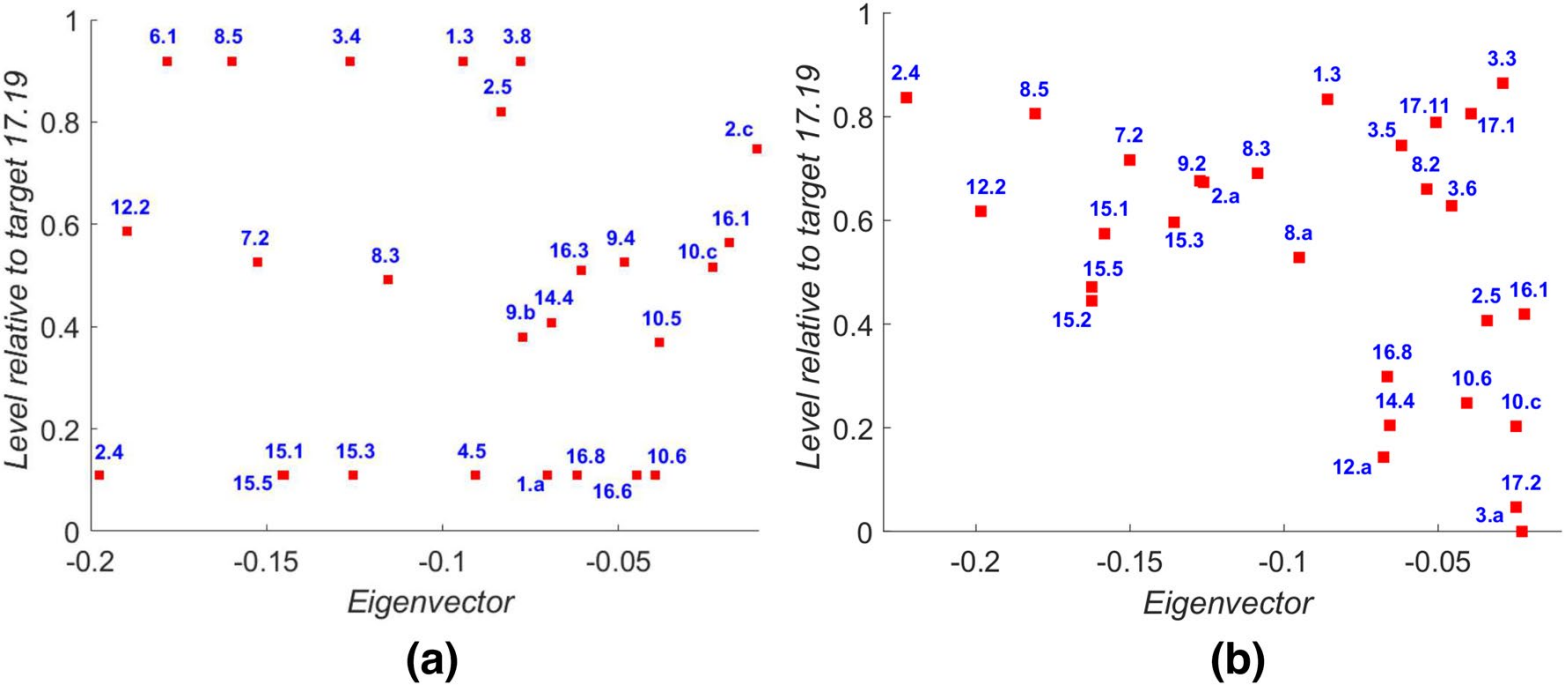
Plots target-by-target of the eigenvector component (horizontal axis) against the level for that target, computed using (9) with $\alpha =-1$. For convenience we set the level $h_i$ for target 17.19 to be zero and plot all other targets relative to this value. Targets that are further to the left have a more negative eigenvector component; targets lower down are further ‘upstream’ in the network. **a** Bangladesh data; **b** Indonesia data

**Fig. 15:**
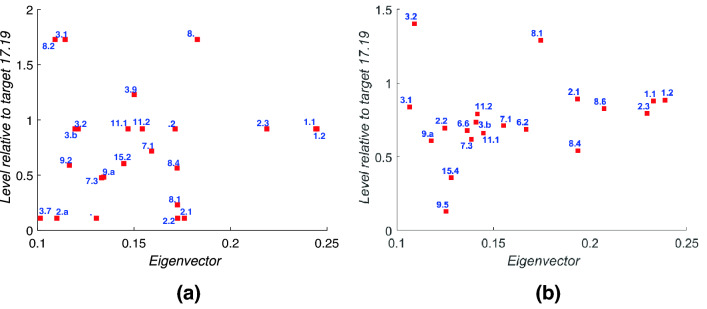
Plots target-by-target of the eigenvector component (horizontal axis) against the level for that target, computed using (9) with $\alpha =-1$. For convenience we set the level $h_i$ for target 17.19 to be zero and plot all other targets relative to this value. Targets further to the right have the highest eigencentrality (component of the leading eigenvector); targets higher up are the furthest ‘downstream’ in the network, and so are expected to benefit most from progress on other targets. **a** Bangladesh data; **b** Indonesia data

Finally we present two pairs of plots for our results that combine the eigencentrality analyses of “Country-level matrices” section with the network hierarchy results. Figure 14 identifies targets that have both low eigenvector centrality and are relatively further upstream in the network hierarchy. These targets are therefore both ‘at risk’ of not being achieved, through network trade-offs and a lack of positive reinforcement effects in the network (shown on the horizontal axis in the plots in Fig. 14) and also are not influenced by progress on other targets since they lie further upstream in the network (vertical axis). Both this figure and Fig. 15 which follows are computed with the exponent $\alpha =-1$, using the definition of ${\tilde{F}}_\alpha (\mathbf {h};A^{\circ \alpha })$ in (8). This was chosen to consider the network in the case that large edge weights bring nodes close together, corresponding to the notion of ‘indivisibility’ in the influence that one has on the other (Nilsson et al. 2016). Details of results for intermediate values of $\alpha$ in the range $-1 \le \alpha \le 1$ are contained in the Electronic Supplementary Material associated with this paper.

We observe that although the details vary between the two cases of Bangladesh and Indonesia there are several targets that appear in both plots, for example 2.4, 7.2, 8.3, 12.2, 14.4, 15.1, 15.3, 15.5, and 16.8.

Figure 15 shows the targets that have both a high eigencentrality, i.e. those on which progress is likely to be most reinforced by the network, and that lie furthest downstream of the others in the network, allowing co-benefits to feed forwards to them. We again observe that, despite the differences between the two countries, a number of targets appear in both plots, including 1.1, 1.2, 2.3, 3.1, 3.2, 3.b, 6.2, 7.1, 8.4, 8.6, 11.1 and 11.2. This points to structural similarities in the targets that we might expect both countries to make most progress on, in the absence of specific policy decisions to support other parts of the SDGs, and again probably reflects the structural constraints provided by the common framework matrix that used in the IGES methdology.

## Discussion and conclusions

We divide this final section into three parts, starting with a summary of our findings, then addressing questions of robustness and the potential for future improvements in the methodology, before finally turning to policy implications.

### Summary

In this paper we introduce mathematical techniques to analyse interlinkage networks building on the target-by-target analysis of the IGES Interlinkages Tool which follows the methodology described in “Data” section and Fig. 1. Among the many attempts to understand SDG interlinkages including ICSU (2015) and ESCAP (2020), the IGES methodology is unique in providing a target-by-target analysis for specific countries through combining expert input, literature reviews and indicator data.

Although the Introduction to the Declaration in the Resolution adopted by the UN General Assembly (UN General Assembly 2015) is clear to point out that the SDGs are a single ‘integrated and indivisable’ agenda, it is equally clear that the wide scope of the SDGs has led many authors (e.g. Le Blanc 2015; Leitner 2017; Independent Group of Scientists 2019) to attempt to find structure within the SDG network. One aim of these analyses, although of course fraught with political issues, is to discern more clearly how to priorities a subset of the SDGs in the hope that a more focussed approach allows more progress across the whole of 2030 Agenda to be made more easily. Putting political and implementation issues to one side, a central methodological question, which this paper addresses, is how to move from the detailed construction of an interlinkage network up to a ‘system-level’ that allows questions of prioritisation to be addressed. The two mathematical tools (Network centrality and network hierarchy) that we present allow the implications for prioritisation of targets within a particular interlinkage network to be deduced.

Our results reveal that for many SDGs, in particular those related to the environment, the average level of network support (‘eigencentrality’) for an SDG disguises the large fluctuations between the support for individual targets. For example within SDG 15 targets 15.1–15.5 score highly in the framework matrix (see Fig. 8) but targets 15.6–15.c all score much lower. The same is true within SDG 12 where target 12.2 (sustainable management of natural resources) stands out at a high value, and in SDG 13 where target 13.1 (resilience to climate-related natural disasters) is highest, and within SDG 14, target 14.2 (the conservation of marine and coastal ecosystems), all of which have relatively high eigencentralities (values of the components of the leading eigenvector of the framework matrix). The framework matrix therefore describes in a broad sense which targets are best connected within the network, but, crucially it does not take into account whether a significant fraction of these connections are in the form of trade-offs rather than co-benefits. For individual countries, as Fig. 6 indicates, the IGES analysis of past time series for SDG indicators reveal many negative correlations.

The ‘network hierarchy’ calculations described in “Network hierarchy” section and results reported in “Network hierarchy” section, show that targets where the leading eigenvector has negative components also generally occur further ‘upstream’ of the others. This implies that these negative influences are indeed able to propagate through the network and act as a drag on the achievement of positive outcomes on targets further ‘downstream’. The negative components of the leading eigenvector would be less of a systematic threat if the targets affected were further downstream in the network themselves but this does not appear to be the case.

Figure 14 summarises the targets that have the combination of being both significantly upstream, in terms of having a low value of the hierarchy statistic, together with a negative component of the leading eigenvector. Note that not all targets are shown in each plot: as we have observed earlier many targets have a zero component of the leading eigenvector as shown in Figs. 9 and 10. Such targets therefore have the greatest potential to prevent achievement of the 2030 Agenda; not only are they unlikely to be achieved themselves but also they are most likely to influence other targets and lead to targets further downstream not being achieved. Targets 2.4, 12.2, 14.4, 15.1, 15.3 and 15.5 appear towards the lower left corner in the plots for both country-specific analyses.

In contrast, Fig. 15 shows that there is also a group of targets that, due to synergies and positive reinforcements through the SDG network are much more likely to be achieved, for both Indonesia and Bangladesh, in particular we identify targets 1.1, 1.2, 2.3, 3.1, 6.2, 7.1, 8.1, 11.1, and 11.2. Since eight of the top 20 are targets within SDGs 1 - 3 we expect that in general those first three SDGs are likely to show the best progress over time.

In the case of target 8.4 which calls for an absolute decoupling of economic growth from resource use and environmental degradation we observe that this target also appears within this group in Fig. 15 for both countries, but we are cautious about whether this implies that it has a strong chance of being achieved since the indicator that is used in our analysis measures only relative improvements in resource efficiency (i.e. material consumption per unit of GDP) rather than absolute improvements. As a result, positive outcomes on target 8.4 could appear to be overstated in our results.

### Robustness of the results

The robustness of our results is impacted by the quality of the framework matrix, the methodology used to quantify interlinkages, and data availability. We comment on each of these in turn.

The framework matrix was developed based on a literature review of work on SDG interlinkages or on specific goals, mainly from United Nations agencies and other regional and international organisations. Limitations or unconscious biases in the selection of references and the review process can impact on the quality of the framework matrix, e.g. by omitting important linkages or including interlinkages that are actually of only minor relevance. Use of different framework matrices will impact on the ranking results (Tables 2, 3) and the hierarchy of the network (Fig. 11). A future direction to improve the systematic review of a large amount of scientific literature and relevant UN documents, would be to use a machine-based text analysis.

Quantification of the country-specific models was based on a correlation analysis of the time-series data for relevant national indicators of the SDG targets. Correlation analysis describes linear relationships between the time series but does not in itself describe causal relationships. The most obvious objections to the use of the linear Pearson correlation statistic $r_{xy}$ defined in (1) are that (i) a strong relationship between the relevant variables may not be captured fully by the statistic if that relationship is not linear, (ii) that even if there is a significant correlation value, this does not indicate a causal relationship since, for example, there may be a common underlying cause that influences both time-series, and (iii) that the underlying association between the time-series may be strong but not show up as a correlation between time-series if it emerges at different times in each series, so that tests for association should take into account lagged values. To deal with the first issue a number of other statistical methods have been proposed, such as the distance correlation measure introduced by Székely et al. (2007), Granger causality analysis (Granger 1988), and the maximal information coefficient (Reshef et al. 2011). An extremely important avenue for future research would be the application of these advanced statistical methods to the underlying time-series to provide robust evidence for these relationships in the historical data. The second point is perhaps more philosophical but could be addressed by the analysis of possible mechanistic explanations for any observed associations that the statistical analyses reveal. The third point can be systematically studied within the frameworks of the statistical methods described above, e.g. Granger causality. Overall these steps would lay significantly more secure foundations for the kinds of system-level analysis that this paper sets out.

Finally, data quality and availability also impacts on the network analysis results. For both Bangladesh and Indonesia, data availability is good for Goals 2 and 3, but particularly poor for Goals 5, 11, 12, 13 and 14 (see Fig. 3). Notably, data is not available for any of the five targets in SDG 13. Since the analysis is at the target level, targets for which indicator-level data is not available are less likely to appear in the highest and lowest-level lists of targets. This lack of data results in the components of the leading eigenvector being zero, as indicated in Figs. 9 and 10. Our ability to comment in detail on targets within those four Goals is therefore compromised. In the case of SDG 5 on Gender Equality this systemic issue is particularly worrying since addressing the data availability issue in respect of SDG 5 demands the collection of gender-disaggregated data across the entire scope of public services and private sector activity. That such data collection is not carried out remains a serious public policy issue and systemic barrier to achieving SDG 5 (Lee and Pollitzer 2020). The robustness of the results is also influenced by indicator quality. Among 231 global indicators, there are 130 Tier I indicators (with defined methodology and data available for at least 50% of countries), 97 Tier II indicators (with defined methodology but data not regularly produced by countries) and 4 indicators with multiple tiers. In the IGES methodology, some proxy indicators and data were used to fill gaps left by global indicators (see Zhou et al. 2021). Data quality and indicator gaps are of course a challenge not only for this study, but are common to many studies of SDG monitoring and quantitative assessment.

### Policy implications

For Indonesia and Bangladesh, the targets with the largest positive components for the leading eigenvector are listed in Table 2 and have significant overlap with those identified in previous studies (Zhou and Moinuddin 2017). These targets enjoy significant co-benefits from progress on other targets and therefore should be those on which most progress is observed. They therefore could be used as targets to monitor to look for the first signs that SDG-related policy interventions are producing the intended results.

Looking at the recent performance of the relevant SDG indicators, it is clear that indicators for nearly all the most promising targets show an upward trend in the past 25 years for both countries. The SDG Interlinkages Tool also shows that these targets have relatively more in-degree linkages with other targets and a larger sum of the correlation coefficients (including both positive and negative ones) over all the in-degree linkages. For instance, Target 2.3 (agricultural productivity) has the most links and the second-largest sum of the correlation coefficients for both countries (114 in-degree links for both countries and the coefficients sum at 16.1 and 16.4 for Indonesia and Bangladesh, respectively). This indicates that these targets receive extensive (more links) and overall net positive support from the system. As a result, poverty reduction (Targets 1.1 and 1.2) and youth employment (Target 8.6) appear in the top part of the rankings. Some differences are also clear. For example, Target 9.2 (inclusive and sustainable industrialisation) appears as one of the promising targets for Bangladesh, which is also evidenced by the data trend over the last few decades. However, the trend of Target 9.2 for Indonesia is regressive and not among the promising targets (in Table 2, it is in fact listed as a target at risk for Indonesia).

Table 2 listing the most promising targets for the two countries clearly reflects national priorities (e.g. poverty reduction, as discussed in “Development context” section) over several decades. Lessons from the successful implementation of these policies should help governments to design similar plans for other targets, particularly those that we identify as being at risk (see Table 3). In addition, policies should aim at leveraging the synergistic effects of the progress across multiple targets.

Targets that have negative components are listed in Table 3. Again, there is significant overlap in the set of targets for both countries on which progress is at risk of stagnating or becoming negative. Trends in the SDG indicators associated with these targets affirm that virtually all of them have either regressed or stagnated over the last 25 years. These targets have the lowest weighted in-degree (which are all negative), i.e. the sum of their correlation coefficients, but their unweighted in-degrees (i.e. the numbers of targets they are connected to) are not necessarily small. To take one example, Target 2.4 (ensure sustainable food production systems) which is represented by Indicator 2.4.1 (fertilisers by nutrient/tonnes) shows a significant negative trend for both countries, implying that achieving target 2.4 is particularly challenging. Target 2.4 has an unweighted in-degree of 102 for both countries, with the sums of the correlation coefficients being $-12.7$ for Bangladesh and $-16.1$ for Indonesia. These overall highly negative in-degree weights illustrate the severity of the trade-offs between this target and other, and need to be addressed to accelerate development; such policy areas should be given specific attention in national SDG planning and implementation. The methodology proposed in the present paper and its empirical applications to Bangladesh and Indonesia will be explored in detail in future work and used to strengthen IGES’ activities in the two countries.

More widely, we note that progress even within SDGs 1–3 is potentially uneven when viewed at the target level, and that targets identified as at risk are particularly present within SDGs 14–16 (e.g. targets 14.4, 15.1, 15.3, 15.5, 16.1 and 16.8), for both countries. There is therefore an overall message that current policy is supporting SDGs 1–3 much more than the environmental and governance aspirations of SDGs 14–16, noting also the lack of indicator data for SDG 13 which compounds this conclusion. This finding is in agreement with the conclusions of previous work (Dawes 2020) and shows the continuing tension within the policy landscape between the human development and environmental preservation aspects of the SDG agenda.

## Supplementary Information

Below is the link to the electronic supplementary material.

Supplementary file1 (PDF 245 KB)
