# Supplementary material for: System-level consequences of synergies and trade-offs between SDGs: quantitative analysis of interlinkage networks at country level
Source: Sustain Sci. 2022 Feb 27;17(4):1435–57. doi: 10.1007/s11625-022-01109-y (PMC8882233; doi:10.1007/s11625-022-01109-y)
Supplement: Supplementary file 1 — Supplementary file1 (PDF 245 KB) [file 11625_2022_1109_MOESM1_ESM.pdf]

Electronic Supplementary Material

System-level consequences of synergies and trade-offs between SDGs: quantitative analysis of interlinkage networks at country level

Jonathan H.P. Dawes*
Centre for Networks and Collective Behaviour and Department of Mathematical Sciences, University of Bath, Bath BA2 7AY, UK

Xin Zhou, Mustafa Moinuddin
Institute for Global Environmental Strategies (IGES), Hayama, Japan

*Corresponding author: `J.H.P.Dawes@bath.ac.uk`

Supplementary Material

This Supplementary Material contains a number of additional figures to illustrate how the results presented in section 3.2 change as the parameter α in equation (8) varies between $+1$ and -1.

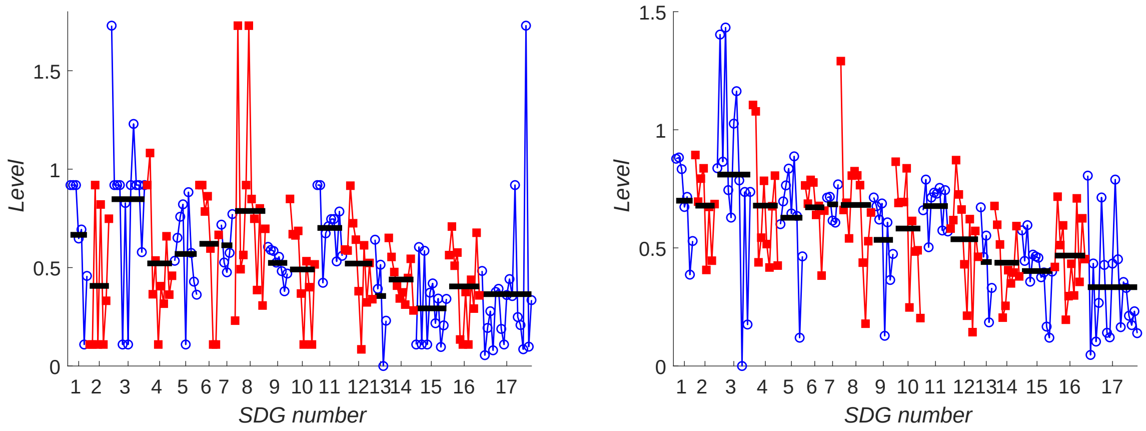

(a) (b)

Figure S1: Relative levels h_i for each target $i = 1, \ldots, 169$, together with averages for each SDG (black horizontal lines) computed for $\alpha = -1$, i.e. strong links imply small levels. (a) Bangladesh matrix $\tilde{A}_{\mathrm{BGD}}$; (b) Indonesia matrix $\tilde{A}_{\mathrm{IDN}}$. Each matrix is regularised by inserting the value $\varepsilon = 10^{-8}$ for those entries where no value is given in the IGES Toolkit but the framework matrix suggests an interlinkage is possible.

Figure S1 plots the levels h_1 in the case $\alpha = -1$, where large values for interlinkages are interpreted as meaning that the two targets are extremely closely related. Although the figure looks at first sight rather different to the case $\alpha = 1$ shown in figure 13 most targets are unaffected by the change in α, as figure S2 shows: the majority of the lines move parallel to each other as α varies: their relative separation does not change.

In the case of Bangladesh, it is interesting to note that target 13.a ('Finance developing countries for mitigation') now occupies the lowest level, albeit with a group of around 25 other targets also now moving to low levels, i.e. $h_i \approx 0.1$.

For Indonesia the target at the lowest level is now 3.a (‘Implement tobacco control convention’) which is one of those highlighted previously in section 3.1.1 as having the lowest in-degree of any target. Target 3.a is also listed in table 3 as having a negative entry in the leading eigenvector for Indonesia (but not for Bangladesh). Indeed, tobacco statistics Drope et al. (2018) also show that smoking remains a much larger public health issue for Indonesia (1676 cigarettes smoked per year per person aged 15 or older, in 2016; ranked 30th in the world) than for Bangladesh (where the corresponding figure for cigarette consumption is 744 (ranked 84th in the world, below the UK and Australia).

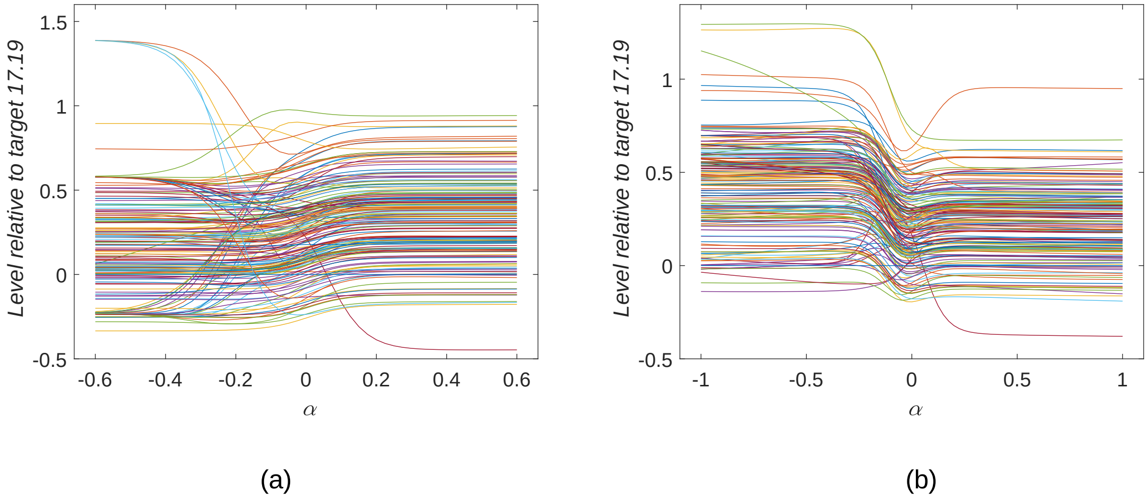

Figure S2: Relative levels h_i for each target $i = 1, \ldots, 169$ as α varies. For convenience we set the level h_i for target 17.19 to be zero and plot all other targets relative to this. (a) Bangladesh matrix A_{BGD} for the range $-0.6 \leq \alpha \leq 0.6$; (b) Indonesia matrix A_{IDN} for the range $-1 \leq \alpha \leq 1$. For values of α outside those shown here the lines remain horizontal indicating that the relative positions of the targets do not change. In both (a) and (b) the dark red line in the lower right is target 8.10.

The robustness of the levels calculations to the value of α for the overall SDGs is brought out by figure S3 which shows that when considered as averages across each SDG, there is only weak dependence of the relative levels of the SDGs on the exponent α: there are few significant cross-overs of the lines within either plot, and indeed the orderings in the extreme cases, shown by the numbered labels at the two sides of each plot, are very similar.

For convenience the levels in figure S3 are shown relative to SDG 17 which is therefore shown as the zero level in both plots. SDGs 1 and 3 are consistently towards the top of the figure and SDGs 13, 15 and 17 are consistently towards the bottom.

In summary this figure supports the view that in terms of the network hierarchy, progress on SDGs 13 (Climate action), 15 (Life on land) and 17 (Partnerships for the goals) has the potential to influence progress on many other parts of Agenda 2030, in contrast to SDGs 1 (No poverty) and 3 (Good health and well-being) which lie much further downstream and do not appear to, in themselves, generate as many co-benefits that enable progress on other parts of the SDG Agenda.

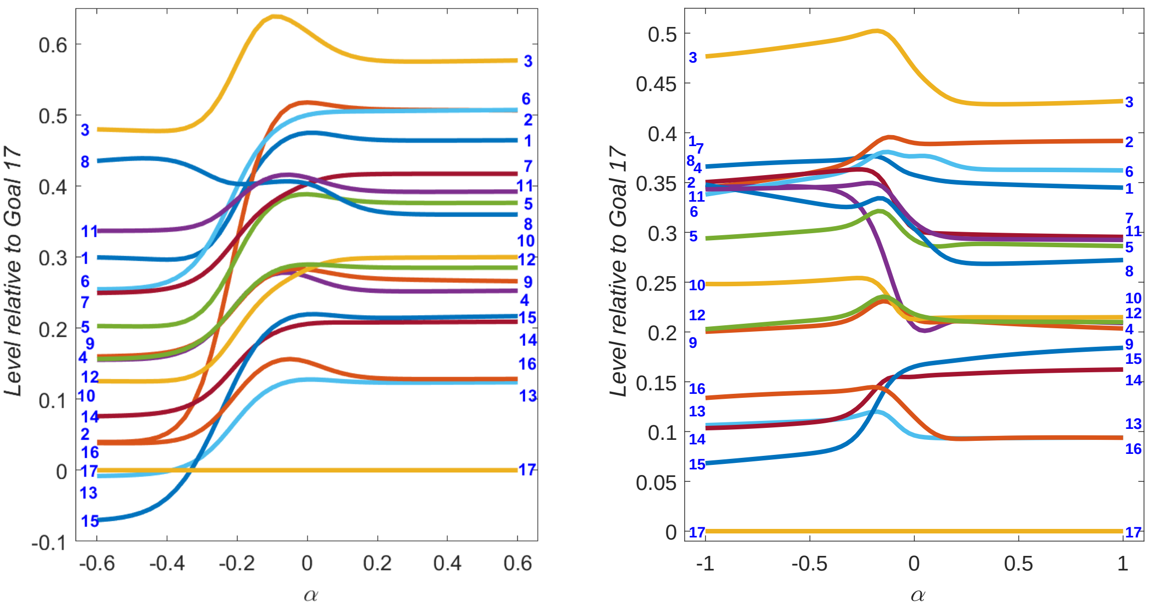

(a) (b)

Figure S3: Relative averaged levels $\bar{h}$ for each SDG as a function of the exponent α. For convenience we set the averaged level for SDG 17 to be zero and plot all other averaged levels for each SDG relative to this. (a) Bangladesh matrix A_{BGD}; (b) Indonesia matrix A_{IDN}. For values of α outside those shown here the lines are horizontal indicating that the relative positions of the targets in the network does not change.

References

J. Drope, N. Schluger, Z. Cahn, J. Drope, S. Hamill, F. Islami, A. Liber, N. Nargis and M. Stoklosa. *The Tobacco Atlas*. Atlanta: American Cancer Society and Vital Strategies (2018)
